# Production, optimization and characterization of esterase isolated from a new endophytic *Trichoderma afroharzianum* strain AUMC 16,433 and its applications in dye decolorization

**DOI:** 10.1186/s12934-025-02832-8

**Published:** 2025-09-09

**Authors:** Yehia A. -G. Mahmoud, Nisrin S. Alamin, Tarek M. Mohamed, Nesma A. El-Zawawy, Maha M. Salem

**Affiliations:** 1 Botany and Microbiology Department, Faculty of Science, Tanta University, Tanta, 31257 Egypt; 2 Biochemistry Division, Chemistry Department, Faculty of Science, Tanta University, Tanta, 31257 Egypt

**Keywords:** Endophytic fungi, Statistical optimization, Purification, Characterization, Dye decolorization.

## Abstract

**Background and aim:**

Synthetic dyes in the textile industry pose risks to human health and environmental safety. The current study aims to examine the efficacy of a novel esterase derived from an endophyte fungus in decolorizing diverse dyes, focusing on its production, purification, optimization, and characterization.

**Results:**

*Trichoderma afroharzianum* AUMC16433, a novel fungal endophyte with esterase-producing ability, was first detected from the cladodes of *Opuntia ficus indica* by ITS-rRNA sequencing. Furthermore, several fermentation variables that augment esterase production were improved by utilising the Plackett-Burman design and RSM. Ammonium sulphate precipitation at 60% and Sephacryl S300 HR gel filtration were employed to purify the isolated esterase to a specific activity of 1372.1 U/mg with a 2.29-fold increase and a recovery of 42.87%. The enzyme’s molecular weight was ascertained to be 43 kDa via SDS-PAGE. The isolated esterase revealed peak activity at 40 °C and pH 8. The kinetic characteristics of esterase were Vmax = 2.717 U/mL and Km = 3.33 mM. The half-life time was 54.4% at 50 °C after 4 h, and the enzyme still retained 14.7% of its activity after 24 h at 50 °C. Esterase decolorized several synthetic dyes used industrially, with the highest decolorization rate in malachite green after 24 h with 66%, and successfully degraded both bromothymol blue and tartrazine with 65.5% and 65.3%, respectively, in the same time frame. Crystal violet and methyl red showed moderate decolorization, with decolorization rates of 57.1% and 43.1%, respectively.

**Conclusions:**

The esterase enzyme isolated for the first time from the new endophytic *Trichoderma afroharzianum* has a high dyes decolorization potential, which offers it a sustainable strategy for addressing environmental contamination issues

**Graphical abstract:**

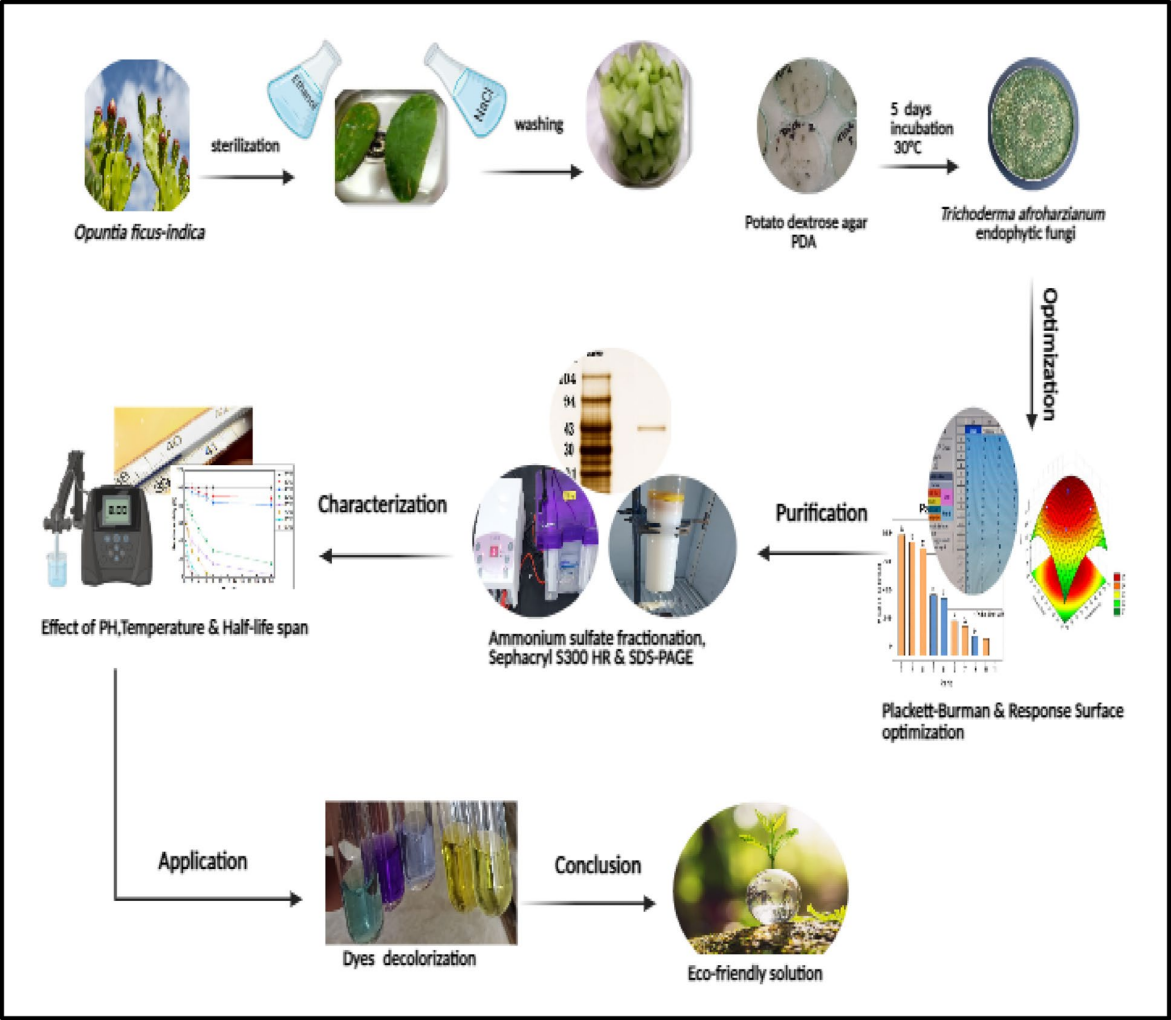

**Supplementary Information:**

The online version contains supplementary material available at 10.1186/s12934-025-02832-8.

## Introduction

Global industrial advancement has intensified the discharge of several detrimental substances into the environment. While sectors are primary drivers of the national economy, their detrimental effluences from manufacturing processes significantly contribute to water pollution [1]. Dyeing textiles, printing on paper, and photographing pictures with colour pharmaceuticals, food, cosmetics, and various other industries all make heavy use of synthetic dyes with an annual output surpassing 700,000 metric tons globally; over 10,000 unique dyes are widely available for commercial use. 10% of the dyes produced are lost during the textile dyeing process; approximately 2% are directly discharged into water [2].

The dyes in industrial effluent adversely affect water quality by obstructing sunlight from penetrating aquatic flora and fauna [3]. Common synthetic dyes include anthraquinone, azo, and triphenylmethane, which are recognized as very detrimental and potentially carcinogenic to certain species. The contamination of wastewater by dyes is an increasing concern due to the extensive use of many colors. Recently, the elimination of color has garnered significant interest from researchers. Microbial biodegradation serves as an environmentally sustainable and economically viable alternative to chemical degradation methods employed for the removal of colored residues in wastewater. While physical and chemical approaches are effective, they can be expensive and generate dangerous by-products [4].

Microorganisms are the principal origin of most enzymes, and it is possible to genetically engineer them to improve enzyme production and enable swift, large-scale proliferation [5]. Phospholipases, lipases, and esterases are hydrolases that catalyze the hydrolysis of substrates like triacyl glycerides, esters, and peptides [5].

Esterases are widely utilized in several industries owing to their stereospecificity, regioselectivity, and stability in organic solvents. Enzymes consistently play essential roles in a wide range of cellular mechanisms, including bioremediation, lipid metabolism, and the hydrolysis of natural chemical substances [6]. Esterases facilitate decolourization, which is advantageous since they transpire under mild conditions at ambient temperature and neutral pH, making them an eco-friendlier and more sustainable alternative to conventional chemical processes [7].

Fungi are regarded as one of the most interesting microbiological sources of commercial enzymes as the remarkable stability, high production potency, and ease of purification and separation operations of fungal enzymes are well-known [8]. Additionally, endophytic fungi have been studied as possible hydrolytic enzyme suppliers [9]. Endophytic fungi are prevalent in plants and reside in their tissues without exhibiting any outward signs of illness [10]. One possible explanation for these fungi’s capacity to produce chemicals with practical industrial, agricultural, and medicinal applications is their existence in plant tissues under harsh conditions [11]. There are no studies about the synthesis of esterase from endophytic fungi, and only *Trichoderma harzianum has* been shown to produce esterase [12]. Therefore, endophytic fungal strains might be a rich biological source that needs to be looked at and studied for the isolation of esterase enzyme.

Prior investigations into dye degradation have underscored various biocatalytic methodologies, notably the employment of oxidative enzymes for the decomposition of complex dye molecules [13]. Whilst the usage of esterases as a distinct class of hydrolase enzymes is comparatively substantial, gaps under investigation include restricted mechanistic insights, a deficiency of application-oriented data, and inadequate investigation of enzyme stability and efficacy under diverse conditions [14]. Furthermore, the potential of new esterase-producing organisms and enzyme optimization techniques remains significantly untapped.

Consequently, our research focused on identifying a novel endophytic fungus from *Opuntia ficus-indica* that produces esterase for the first time, optimizing the enzyme’s production conditions, purifying it, characterizing its biochemical properties, and assessing its efficacy in the decolorization of synthetic dyes, thereby advancing sustainable environmental management.

## Materials and methods

### Chemicals and dyes

The current study used potato dextrose agar (PDA), (Ifco Laboratories, Detroit, MI, USA), maltose (Srichem, India), glucose, sodium chloride, ammonium sulphate (cat# A4418), and peptone (cat# P6838) were purchased from (Merck, USA). Coomassie brilliant blue R250 (CBB), sephacryl S300 HR, P-nitro phenyl acetate (p-NPA), P-nitro phenol, tris-hydrochloride, sodium lauryel dodecyl sulphate, and bromophenol blue were obtained from (Sigma Aldrich, Germany). Bovine serum albumin (BSA), sodium carbonate, sodium hydroxide, and acetate buffer, malachite green 90% (CAS#569-64-2), crystal violet (CAS#548-62-9 ), bromothymol blue(CAS#76-59-5), methyl red(CAS#493-52-7), light green SF yellowish 70% (CAS #5141-20-8), tartrazine 85% (CAS#1934-21-0), cresol red 95% (CAS# 1733-12-6), methylene blue 70% (CAS# 122965- 43-9) and direct fast turquoise blue 95% (CAS# 1330-38-7) was purchased from Qingdao Sanhuan Colorchem Co., Ltd.

### Source of fungi

Five different endophytic fungi (EF) were designated as (EF-1, EF-2, EF-3, EF-4 and EF-5) which were previously isolated from the cladodes of *Opuntia ficus-indica* (L.) Mill. (Cactaceae) were used in this study [15]. Strains were cultivated on petri dishes comprising potato dextrose agar medium, which is fortified with chloramphenicol (300 µg/mL) to hinder the growth of any bacterial species. After 5 days of incubation at 30 °C, the plates were kept at 4 °C until subsequent application.

### Screening of esterase-producing EF

One hundred microliters of each selected endophytic fungal strain suspension (2 × 10^5^) combined with 100 µL phosphate buffer, 20 µL substrate, and 6 µL pH indicator. After four days of incubation at 28 °C, spectrophotometric analysis revealed a color shift in the reaction medium. The process yielded carboxylic acids, namely acetic acid. Utilizing a concentration of 2 µL/mL of acetic acid served as a positive control. A buffer solution was a negative control [16, 17].

### Identification of positive esterase EF

Conventional morphological characteristics were employed to detect positive esterase EF. Two characteristics of the colonies were observed: density and aerial mycelium. A light microscope (Olympus CX51, Japan) was employed to investigate the micromorphological characteristics utilizing the methods previously outlined [18].

Cultures were dispatched to the Molecular Biology Research Unit at Assiut University for DNA extraction utilizing the path-gene spin DNA/RNA extraction kit provided by the Korean company Intron Biotechnology, employing molecular techniques to verify the identification of a specific endophytic fungal isolate. The SolGent Company, located in Daejeon, South Korea, obtained fungal DNA samples for PCR and rRNA gene sequencing. Upon incorporation into the reaction mixture, the ITS1 (forward) and ITS4 (reverse) primers were employed for PCR. The ITS1 (5′-TCC GTA GGT GAA CCT GCG G-3′) and ITS4 (5′-TCC TCC GCT TAT TGA TAT GC -3′) primers are as follows. For sequencing of the amplified PCR product, dNTPs and the same primers were included in the reaction mixture [19]. The recovered sequences were aligned with the Basic Local Alignment Search Tool (BLAST) provided at the National Centre for Biotechnology Information (NCBI) website. The recovered sequences were compared by BLAST analysis with the NCBI database for similarity with closely related organisms. A neighbor-joining phylogenetic tree of the ITS rRNA gene was generated with the MEGA X software [20].

### Production of esterase

The growth medium used for esterase enzyme production was maltose (1.0 w/v), cottonseed oil (4.0 v/v), peptone (2.0 w/v), and sodium chloride (0.5 w/v). The medium was assumed to be sterile after 20 min of autoclaving at 121 °C and 1.5 atm. One hundred milliliters of the sterilized medium was injected with 100 µl of fungal culture and incubated at 40 °C for six days [21]. After centrifugation, the filtrate served as the enzyme solution.

### Esterase assay

p-Nitrophenyl acetate (p-NPA) is used as a substrate to evaluate the activity of esterase enzymes. The activity of the enzyme was quantified as the quantity of the esterase enzyme that liberates 1 µmol of p-nitrophenol per minute at 37 °C [23]. The assay solution comprised 600 µL of Tris buffer (50 mM, pH 8), 40 µL of p-nitrophenyl acetate (5 mM), 200 µL of an enzyme (fungal extract), and 160 µL of distilled water, which was then incubated at 37 °C for 10 min. The reaction was stopped by placing the mixture in the freezer at -20 °C for two minutes. The measurement was conducted at a specific wavelength of 410 nm. The Bradford method has been used in measuring protein content, using bovine serum albumin (BSA) as the reference standard [22].

### Optimization parameters for positive esterase EF

#### Evaluation of critical parameters with Plackett-Burman design

The Plackett-Burman design is primarily used for identifying critical factors influencing process output metrics. Diverse experimental combinations are employed to mitigate the influence of inexplicable fluctuation in actual esterase activity attributable to extraneous variables [23, 24]. The design was implemented for evaluating several production variables for esterase activity at two levels: minimum (^_^1) and maximal (+ 1), as shown in Table (S1).

The research examined six variables: cottonseed oil content, peptone concentration, maltose concentration, pH, temperature, and inoculum size. Plackett-Burman and regression analyses were conducted using statistical software Design-Expert 7.0 (Stat Ease Inc., Minneapolis, U.S.A). The experiment was carried out in 12 runs to investigate the effect of the specified variables on esterase activity. All trials were done in triplicate, and the average of esterase activity was used as the response. The statistical significance of the first-order model was determined using Fisher’s test for analysis of variance (ANOVA) [25]. Plackett–Burman experimental design is based on the first order model:

1 $${\text{Y=}}\beta 0+\sum {\beta iXi} $$

where, Y is the response or dependent variable (esterase activity); it will always be the variable we aim to predict, β0 is the model intercept and βi is the linear coefficient, and Xi is the level of the independent variable which will help us explain esterase activity.

### Central composite design (CCD)

The ideal values of key variables were determined to enhance esterase yield from the selected EF. The significant independent variables in the Plackett-Burman experiment were then optimized by CCD to ascertain the optimal values and interactions of the variables [26], as demonstrated in Table (S2) The cumulative impact of many variables on esterase yield was assessed by generating 3-D response curves for two independent parameters while maintaining other independent parameters at their baseline levels. The model’s significance and regression coefficients were determined by analysis of variance (ANOVA). The coefficient of determination (R²) was utilised to evaluate the appropriateness of the polynomial equation. After establishing the optimal parameter values, three-dimensional plots were employed to assess the impact of a specific set of independent variables on the esterase yield from the selected EFs [27]. A total of 17 experimental sets were made to fit a full quadratic equation model. The equation was given as below:

2 $${\text{Y }}={\text{ }}{\beta _0} - \sum {\beta _{\text{j}}}{{\text{X}}_{\text{j}}}+\sum {\beta _{{\text{j j}}}}{{\text{X}}_{\text{j}}}^{{\text{2}}}+\sum \sum {\text{ }}{\beta _{{\text{ij}}}}{{\text{X}}_{\text{i}}}{{\text{X}}_{\text{j}}}$$

where, Y represented dependent variable; 0, j, jj, and ij were the regression coefficients for intercept, linearity, square, and interaction, respectively; Xi and Xj were the independent coded variables; and k represented the number of variables. The actual data and predicted responses were analysed using Design-Expert 13.0. Analysis of variance (ANOVA) was performed, and three-dimensional surface plots and contour plots were used to calculate the optimal fermentation conditions of esterase production by the selected isolate.

### Validation of statistical model

To validate the model’s validity, accuracy, and stability, one predicted trial estimated using the CCD numerical optimization was chosen as a checkpoint and put through an experimental test to determine the deviation percentage. A comparison of esterase production before and after optimization (in the ideal circumstances predicted by the model) was done to further validate the outcomes produced using CCD.

### Purification of esterase

The crude esterase was purified using 60% ammonium sulfate fractionation. The precipitate fraction was centrifugation at 5000 rpm for 10 min at 4 °C to separate and collect the protein. The smallest volume of 50 mM tris-HCl buffer, pH 8.0, was used to dissolve the precipitate [16, 28]. Next, A Sephacryl S300 HR gel filtration chromatography (GFC) apparatus was employed and positioned vertically on an appropriate ring holder. The column (20 × 3 cm) was washed multiple times with distilled water, then 2–3 times with 50 mM tris-HCl buffer at pH 8.0 to pack and equilibrate it with the buffer. The amount of buffer solution used for elution was 2 volumes of the resin. 3 mL of partially purified esterase enzyme was loaded after ammonium sulfate precipitation on the column. A flow rate of 3 mL/5 min was used for elution, and the protein content of the fractions was evaluated using UV-Vis spectrometry, which assessed absorbance at 280 nm [29].

### Molecular mass polyacrylamide gel electrophoresis using sodium dodecyl sulfate (SDSPAGE)

Following the methodology a 10% (w/v) acrylamide gel was used for SDS-PAGE in a 10x Tris-glycine buffer at pH 8.3. Biorad Clever Scientific OmniPAGE Mini electrophoresis equipment was used. The protein was stained with silver nitrate as the method of Wray et al. [30].

Clean surfaces were used to assemble glass plates clamped into a casting frame. Fresh separating gel (5 mL) was added to glass plates and polymerized for 30–45 min. Next, the stacking gel was placed above the separating gel, and a comb was inserted to produce wells. Polymerization in the gel was permitted for 20 min. Afterwards, protein samples were prepared by adding 5µL sample buffer to 20 µL of purified esterase and heating at 90 °C for 3 min, then applied to the gel. After adding the running buffer, electrophoresis was powered at 80 volts for 30 min, then 100 volts for 1 h. After electrophoresis, the power was turned off, and the gel plates were removed with care. For silver staining, the gel was rinsed with distilled water and subsequently immersed in a silver nitrate solution (0.8 g AgNO₃ dissolved in 4 mL H₂O) for exactly 20 min on a shaker. After staining, the gel was washed twice with H₂O for 10 min each. To develop the stain, the gel was transferred to the destaining solution (2.5 mL of 1% citric acid and 250 µL of formaldehyde, and the final volume was adjusted to 500 mL with distilled water, where protein bands appeared within 10–15 min. Finally, the reaction was stopped by immersing the gel in 1% acetic acid [30].

### Biochemical characterization of purified esterase activity

#### Effect of temperature

The reaction mixture was incubated at various temperatures from 20 to 90 °C using standard assay protocols as previously mentioned to identify the optimal reaction temperature for pure esterases. The remaining enzyme activities were subsequently evaluated using the aforementioned approach [31].

### Effect of pH

Various pH values were examined for isolated pure esterases utilising p-NPA as a substrate, within the range of 3.6 to 10, under conventional assay conditions. The pH of pure esterases was adjusted using different buffers such as 50 mM acetate buffer at pH 3.6, 4.0, and 5.6, Tris-HCl (50 mM) at pH 7.0, 8.0, 9.0, and 10 [31].

### Kinetic parameters

#### Determination of Km and vmax

Pure esterase activity towards various p-nitrophenyl acetate concentrations from 1 to 10 mM was evaluated, under conventional assay conditions. The reciprocal plot graphic method devised by Lineweaver-Burk in 1934 was used to compute the Michaelis-Menten constant (Km) and maximal reaction velocity (Vmax) [32].

### Evaluation of half-life span

The stability and activity of pure esterase were assessed by determining its half-life span at a range of temperatures (20 °C to 90 °C) through activity measurements at multiple time intervals, including 30 min, 1 h, 2 h, 4 h, 6 h, 8 h, and up to 24 h. The decrease in activity over time at each temperature was used to calculate the half-life span, indicating the duration for the enzyme to lose 50% of its initial activity. The esterase activity was measured under the previously specified standard assay conditions [33].

### Bioremediation application of pure isolated esterase in dye decolorization

Decolorization of dyes was carried out using the purified *Trichoderma afroharzianum* esterase on nine different dye types as shown in Table (S3), which were malachite green, crystal violet, bromothymol blue, methyl red, tartrazine, direct fast turquoise blue, light green SF yellowish, cresol red, and methylene blue. Briefly, dye solutions (50 mg/mL) were prepared by dissolving each dye in 50 mM Tris-HCL buffer at pH 8.0. The reaction mixtures were incubated with pure esterase at 40 °C for 30 min, 1 h, 2 h, and 24 h. In the same conditions, the dyes without the enzyme were incubated as a control. Decolorization was determined by measuring the absorbance (OD) of both sample and control at the wavelength specific to the dye using a spectrophotometer [34]. The percentage of decolorization was calculated as mentioned in the following Eq. (3) .

3 $$ \begin{aligned} \:{\text{Decolorization}}\:{\text{percentage}}\:\% &= \:\frac{{{\text{Control}}\:\left( {{\text{OD}}} \right)\:{\text{ - sample}}\left( {\:{\text{OD}}} \right)\:}}{{{\text{Control}}\:\left( {{\text{OD}}} \right)}} \\ & \times \:{\text{100}} \\ \end{aligned} $$

### Statistical analysis

The studies were done three times, and the results were given as the mean ± SD for all three times. Significant differences were found using a one-way ANOVA. Differences were significant when *p* < 0.05.

## Results and discussion

Advanced industrialization has led to an escalation in the continuous discharge of detrimental effluents into the environment. This is particularly applicable to industrial facilities that generate significant volumes of wastewater containing persistent colors. The synthesis of novel esterases originating from microbes is a crucial step for numerous biotechnological applications requiring the development of stable and cost-effective biocatalysts [31].

Consequently, this study examined bioremediation methods utilizing microbial esterase due to their ecological advantages and economic efficiency. Esterase is an enzyme involved in several processes, including waste detoxification and dye decolorization. Thus, our research concentrated on esterases generated from endophytes and their prospective applications in dye decolorization.

### Detection and identification of EF strains with maximal esterase production

To assess esterase enzyme production, an initial screening was performed on five endophytic fungal strains (EF-1, EF-2, EF-3, EF-4, and EF-5) (Fig. S1). Following six days at 28 °C, only the EF-2 strain exhibited positive esterase activity, whilst the other strains demonstrated no potential for esterase synthesis. Consequent to these findings, the EF-2 strain was chosen for morphological and molecular identification, subsequent esterase extraction and characterization, and ultimately, for esterase production.

**Fig. 1:**
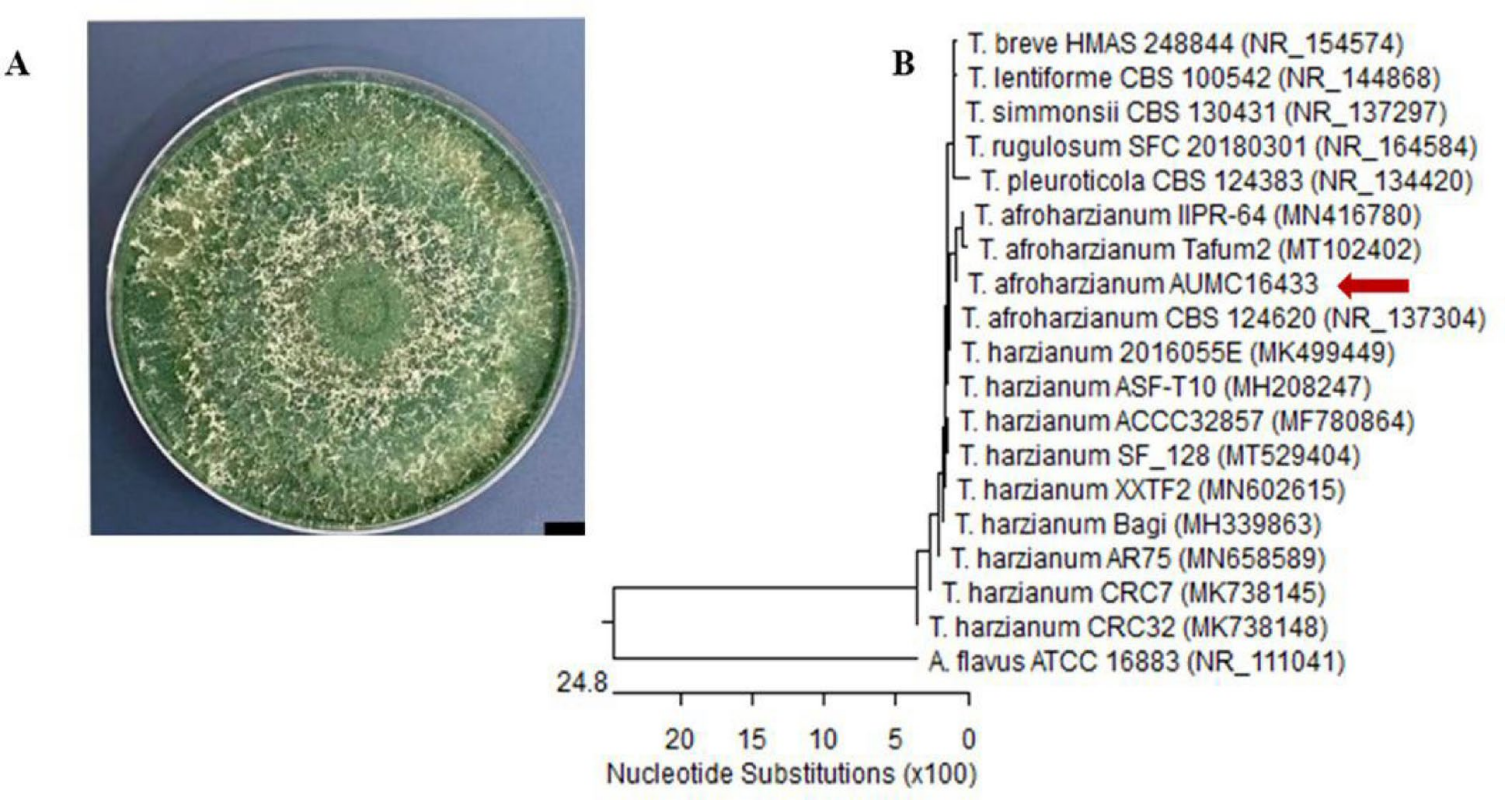
Morphological and molecular identification of esterase-producing fungal endophyte. **A**. Colonial morphology of the positive esterase-producing fungi endophytes, **B**. A phylogenetic tree was generated using ITS sequences of rDNA of esterase-producing fungal endophyte (*Trichoderma afroharzianum* AUMC16433; GenBank accession number PQ221217)

Colonies exhibiting white, whitish dark, and dark green hues were observed in (Fig. 1A). Microscopic examination revealed short, robust phialides with numerous branches, green, oval-shaped, thick-walled conidia, and hyaline, insulated hyphae. Identification of this strain was achieved through ITS-rDNA gene PCR and morphological data (Fig. 1B), EF-2 strain is *Trichoderma afroharzianum* strain AUMC 16,433, with GenBank accession number PQ221217. It encompasses several *T. afroharzianum* strains, including the type of strain CBS1245290 (GenBank accession NR_137304). The sequenced strain exhibited 99.66%-100% similarity with *T. harzianum*. The ITS region (Internal Transcribed Spacer) is a highly conserved segment located within the rDNA gene of fungi. This location has been extensively utilized for the molecular identification of fungal species by PCR-based methodologies, encompassing PCR amplification and agarose gel electrophoresis [35, 36]. The same results have been shown in an investigation conducted in 2022 [37].

### Evaluation of key parameters using the Plackett-Burman design

Twelve distinct experiments were conducted employing the Plackett-Burman design to evaluate the impacts of six factors on our study witch, inoculum size, pH, peptone, maltose, cotton seed oil, and various combinations as detailed in Table (1), which encompassed various combinations of factors. The results reveal a remarkable increase of 165.5 to 418.5 U/ml in esterase yield.

**Table 1:** Placket-Burman experimental design for esterase production by *T. afroharzianum*

Run	Cotton seed oil (%, v/v)	Peptone (%, w/v)	Maltose concentration (%, w/v)	pH	T (◦C)	Inoculum size (%, v/v)	Response (OD)	Esterase activity Per min	Esterase activity Per hour
1	1	1	1	-1	-1	-1	0.523	2.758	165.5
2	-1	1	1	-1	1	1	0.635	3.69	221.5
3	-1	1	1	1	-1	-1	1.029	6.975	418.5
4	1	-1	1	1	1	-1	0.773	4.841	290.5
5	-1	-1	-1	1	-1	1	0.654	3.85	231
6	1	-1	-1	-1	1	-1	0.688	4.13	248
7	1	1	-1	-1	-1	1	1.100	7.56	454
8	1	-1	1	1	-1	1	0.850	5.48	329
9	-1	-1	1	-1	1	1	0.769	4.80	288.5
10	-1	-1	-1	-1	-1	-1	0.829	5.30	318.5
11	-1	1	-1	1	1	-1	0.728	4.46	268
12	1	1	-1	1	1	1	1.005	6.77	406.5

**Fig. 2:**
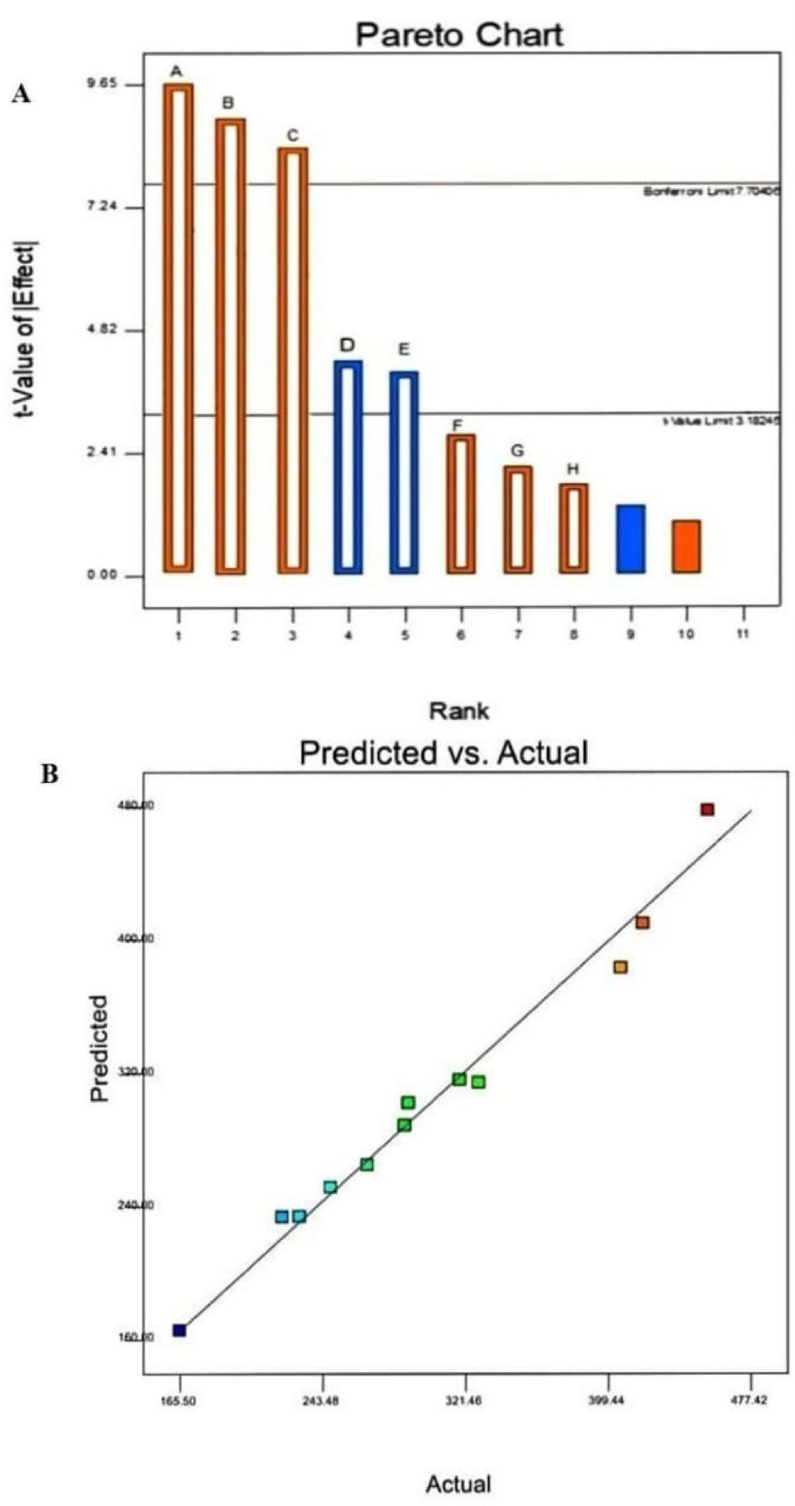
Pareto chart depicts the degree to which each variable influences esterase production by *T. afroharzianum*, **A**. Correlation between the experimentally actual and predicted values for esterase activity by *T. afroharzianum* according to the Plackett–Burman experimental results **B**

The results were used to prepare a Pareto chart (Fig. 2) to identify the importance of the factors that affect esterase production from *T. afroharzianum.* Peptone, cottonseed oil, and maltose were the most effective three critical parameters in esterase production in our study. Following our findings, Plackett-Burman results from *Serratia* sp. for esterase production increased from 0.331 to 6.02 U/ml. Giving peptone, cottonseed oil, and maltose are the most important parameters for esterase production [38].

### Response surface by central composite design

Response Surface Methodology (RSM) is a technique commonly employed to investigate the impact of many variables and to optimize different biological processes [40]. Various statistical models can be utilized to test the interaction effects of a parameter on enzyme yield so that the optimal variable outcome can be computed [39]. The industrial application implements RSM mainly to decrease the experimental runs, as it is less labour-consuming and more effective [40]. RSM allows for the study of several variables on different levels at a time in order to determine their joint effects, hence leading to efficient optimization results [41].

In our study, seventeen distinct experimental setups were designed for the three most significant positive parameters using CCD, and the outcomes of various experimental responses (esterase yield) were recorded as shown in Table (2). In run 17, the maximum enzyme activity was attained (480.50 U/mL). This aligned with the expected value (473.37 U/mL), indicating that the model fitted was strong, as in Table (3). The ideal conditions for esterase synthesis included (3.0%, v/v) cottonseed oil, (2.0%, w/v) peptone, (1.0%, w/v) maltose, a pH of 7.0, a temperature of 28 °C, and an inoculum size of (1.5%, v/v), optimal conditions for esterase synthesis. The analysis of variance (ANOVA) to produce esterase by *Trichoderma afroharzianum* in Table (4) shows that the model is statistically significant (F-value = 4.87, p-value = 0.0244).

**Table 2:** Analysis of variance (ANOVA) and regression statistics for the experimental results of Plackett Burman design of esterase production by *T. afroharzianum*

Source	Sum of Squares	df	Mean Square	F-value	*p*-value	
Model	81418.33	8	10177.29	17.97	0.0185*	significant
A- Cotton seed concentration	1813.02	1	1	1813.02	3.20	0.1716
B-Peptone concentration	4351.02	1	1	4351.02	7.68	0.0695
C-Maltose concentration	10049.20	1	1	10049.20	17.74	0.0244
D-pH	52717.70	1	93.06	0.0024	0.0279*	
E-Temp.	9089.70	1	9089.70	9089.70	0.0230*	
F-Inoculum size	10520.50	1	10520.50	10520.50		
Residual	1699.40	3	566.47			
Cor Total	83117.73	11	R- Squared	0.9796		
Std. Dev.	23.80		Adj R-Squared	0.9250		
Mean	303.2		Adeq Precision	15.133		
C.V%	7.85					

**Table 3:** Seventeen trials of central composite design representing esterase production by *T. afroharzianum*

Run Order	Cotton seed oil concentration (%, v/v)	Peptone concentration (%, w/v)	Maltose concentration (%, w/v)	Esterase activity (U/mL)	
				Actual Value	Predicted Value
1	0	0	0	159.50	154.9
2	1	-1	0	70.50	128.44
3	-1	0	-1	312.00	298.50
4	0	1	-1	91.50	162.94
5	-1	-1	0	206.50	273.56
6	0	1	1	123.00	176.56
7	0	-1	1	267.00	195.56
8	-1	0	1	469.00	422.26
9	1	1	0	167.00	99.94
10	0	0	0	166.50	154.93
11	1	0	1	144.00	157.50
12	0	0	0	149.50	154.99
13	0	0	0	149.50	154.80
14	0	0	0	149.50	154.90
15	0	-1	-1	77.00	23.44
16	1	0	-1	151.00	146.6
17	-1	1	0	480.50	473.37

**Table 4:** ANOVA of the fitted quadratic polynomial model of esterase from *T. afroharzianum*

Source	Sum of Squares	df	Mean Square	F-value	*p*-value	
Model	2.022	9	22466.37	4.87	0.0244	significant
A-Cotton seed concentration	1.094	1	1.094E + 05	23.71	0.0018	
B-Peptone concentration	7260.13	1	7260.13	1.57	0.2500	
C-Maltose concentration	17251.53	1	17251.53	3.74	0.0944	
AB	7876.56	1	7876.56	1.71	0.2327	
AC	6724.00	1	6724.00	1.46	0.2666	
BC	6280.56	1	6280.56	1.36	0.2815	
A²	44496.17	1	44496.17	9.64	0.0172	
B²	2973.60	1	2973.60	0.6445	0.4485	
C²	537.64	1	537.64	0.1165	0.7429	
Residual	32298.76	7	4614.11			
Lack of Fit	32055.56	3	10685.19	175.74	0.0001	significant
Pure Error	243.20	4	60.80			
Cor Total	2.345	16	**R²**	0.8623		

**Fig. 3:**
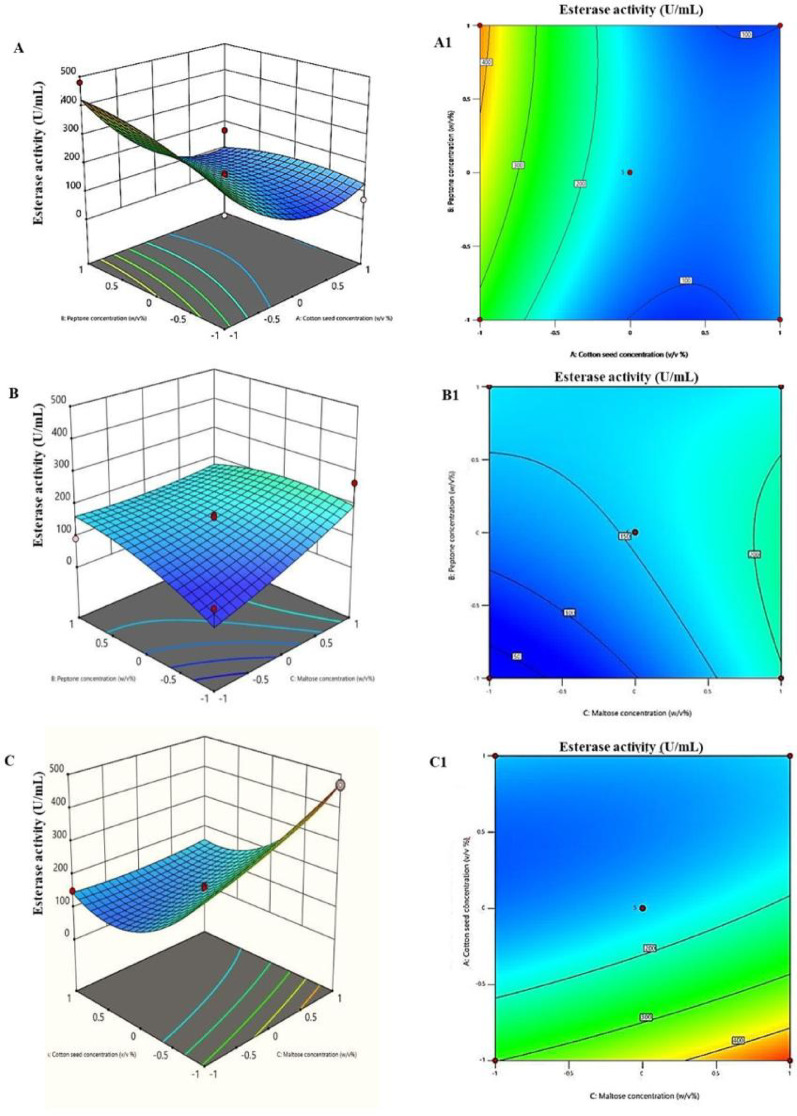
The 3D surface response and contour plots showing the mutual interaction effect of cotton seed oil concentration; peptone concentration; and maltose concentration on esterase production

**Fig. 4:**
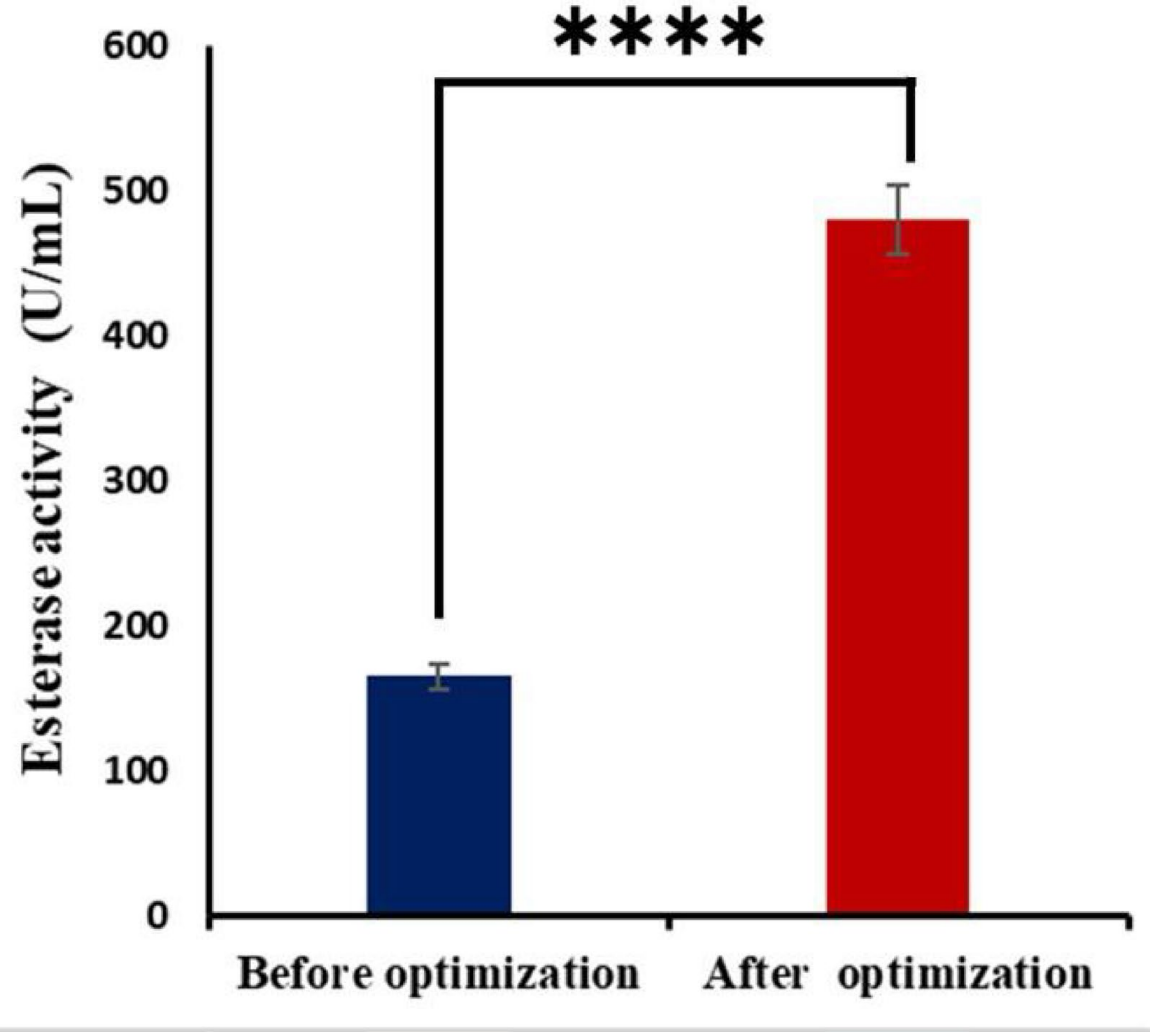
Esterase activity before and after RSM optimization

The 3D surface plots (Fig. 3A, B, and C) and their corresponding contour plots (Fig. 3A1, B1, and C1) illustrated the interactions between each pair of variables affecting esterase activity of *T. afroharzianum*, while holding the third variable constant. (Fig. 3A, 3A1) illustrated the interaction between cotton seed and peptone concentrations. The surface and contour plots reveal a curved relationship, suggesting a significant interaction. Higher esterase activity appears at low peptone and high cotton seed concentrations. (Fig. 3B, 3B1) showed the interaction between peptone and maltose concentrations. The relatively flat surface indicates a weaker interaction, with esterase activity decreasing slightly as both concentrations increase. (Fig. 3C, 3C1) represented the interaction between cotton seed and maltose concentrations. A strong positive interaction is observed here, with esterase activity increasing significantly at higher concentrations of both components. Overall, these plots identified optimal conditions for maximizing esterase activity. The most promising combination appears to be high cotton seed and maltose concentrations, while peptone shows a less favorable influence. After applying the numerical optimization design, the yield of esterase increased 2.9-fold as compared to the yield before the entire optimization step as in (Fig. 4).

Similarly, in a recent study in 2021, it was determined that 0.3% maltose had the highest esterase enzyme activity (13.25 U/mL), following the administration of 0.5% maltose, enzyme output diminished to 4.58 U/mL, and a concentration of 0.4% maltose yielded a similar outcome [42]. Using a fractional factorial central composite design, esterase as one of the lipolytic enzymes was previously produced by *Enterobacter aerogenes*, showed optimum activity at a temperature of 34 degrees Celsius, pH of 7.0, inoculum volume of (2.0%, v/v), oil concentration was (3.0%, v/v) and an incubation time of 60 h [43].

### Purification of T. afroharzianum esterase enzyme

The esterase produced by *T. afroharzianum* strain AUMC 16,433 in the culture fluid under ideal growth conditions and optimized medium composition was purified through several methods. Initially, 60% ammonium sulfate fractionation was applied to the crude esterase enzyme, followed by further purification using Sephacryl S300 HR gel filtration chromatography.

**Fig. 5:**
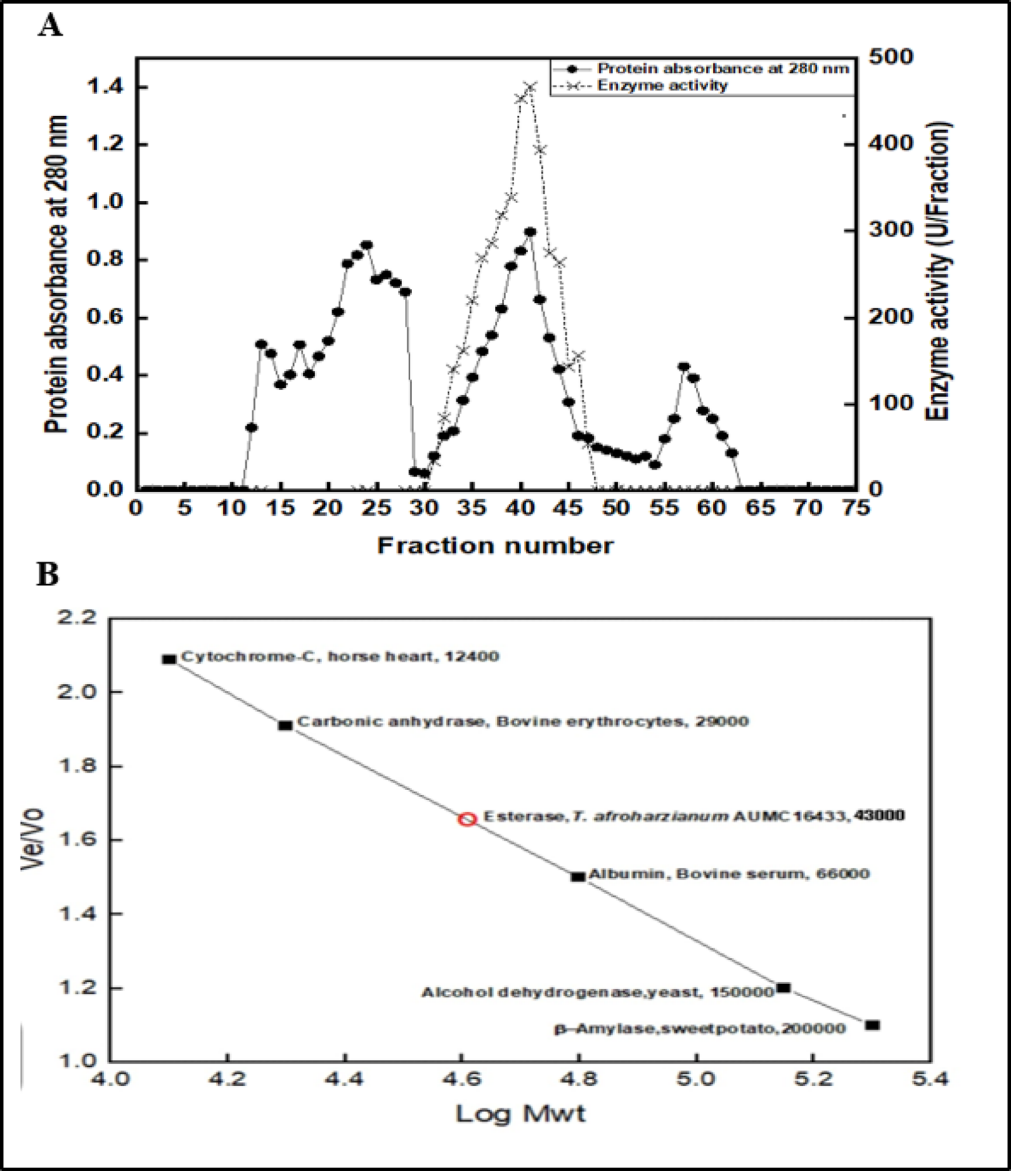
**A**. Elution profile of purified esterase from *T. afroharzianum* using Sephacryl 300 HR, **B**. The molecular weight of purified esterase from *T. afroharzianum* using Sephacryl S300 HR and standard protein markers which are cytochrome-C, horse heart (12.4 KDa), Carbonic anhydrase, Bovine erythrocytes (29 KDa), Albumin, Bovine serum (66 KDa), Alcohol dehydrogenase, yeast (150 KDa) and β-amylase, sweet potato (20 KDa)

The outcomes of these procedures were thoroughly documented in Table (5). Our findings revealed that after ammonium sulfate fractionation, the fold purification was 1.7-fold and specific activity increased from 597 to 1036.3 U/mg, with a recovery rate of 71.2%. Further purification via the Sephacryl S300 HR gel filtration column resulted in a maximum specific activity of 1372.1 U/mg, representing a 2.29-fold increase and 42.87% recovery as represented in **(**Fig. 5A). Other previous studies showed that the esterase enzyme extracted from *Aspergillus niger* exhibited a specific activity of 114.20 U/mg, with a purification fold of 47.58 [41]. The isolated and purified intracellular esterase from *Bacillus aryabhattai* B8W22 achieved a purification fold of 59.03 and a yield enhancement of 20% [44]. A study in 2016 demonstrated an 8.93% yield, a specific activity of 8121.51 U/mg, and a purification fold of 9.33 in their account of esterase enzyme purification [47].

**Table 5:** Purification profile of esterase isolated from *T. afroharzianum*

Steps	Total activity (U)	Total protein (mg)	Specific activity (U/mg)	Fold of purification	Recovery (yield %)
Parameter					
Crude isolate of T. afroharzianum esterase	8000	13.4	597	1	100%
60% Ammonium sulphate fractionation	5700	5.5	1036.3	1.7	71.25%
Sephacryl 300 h	3430.2	2.5	1372.1	2.29	42.87%

(Fig. 5B). Indicates that the molecular weight of the esterase enzyme isolated from *T. afroharzianum* strain AUMC 16,433 was 43 kDa. Esterase molecular weight varies depending on its kind of acetyl, glucuronoyl or carboxyl, as well as its source, mentioned in many studies.

Previous studies showed that the *Bacillus mojavensis* strain TH309 esterase is 30 kDa by using ultrafiltration and anion-exchange techniques [31]. A study from India reported that the molecular weight of the esterase enzyme derived from *Aspergillus Versicolor* was 32 kDa, determined using Sephadex G-75 [16]. The molecular weight of the purified esterase from *Bacillus licheniformis* by using Sephadex G-75 column chromatography was 42 kDa [45]. Previous studies indicated that the esterase extracted from *Enterobacter cloacae* possessed a molecular weight of 54 kDa, as determined by high-performance liquid chromatography [46]. The enzyme esterase isolated from *Trichoderma ressi* showed a molecular weight of 67 kDa determined by using Sephacryl S-200 SF column gel filtration [47]. The molecular weights of the two esterase enzymes isolated from the *parasite Fasciola gigantica* using Sephacryl 300 gel filtration chromatography were 50 KDa and 56 KDa, respectively [51].

### Evaluation of the purity and integrity of the purified T. afroharzianum esterase using SDS-PAGE

**Fig. 6:**
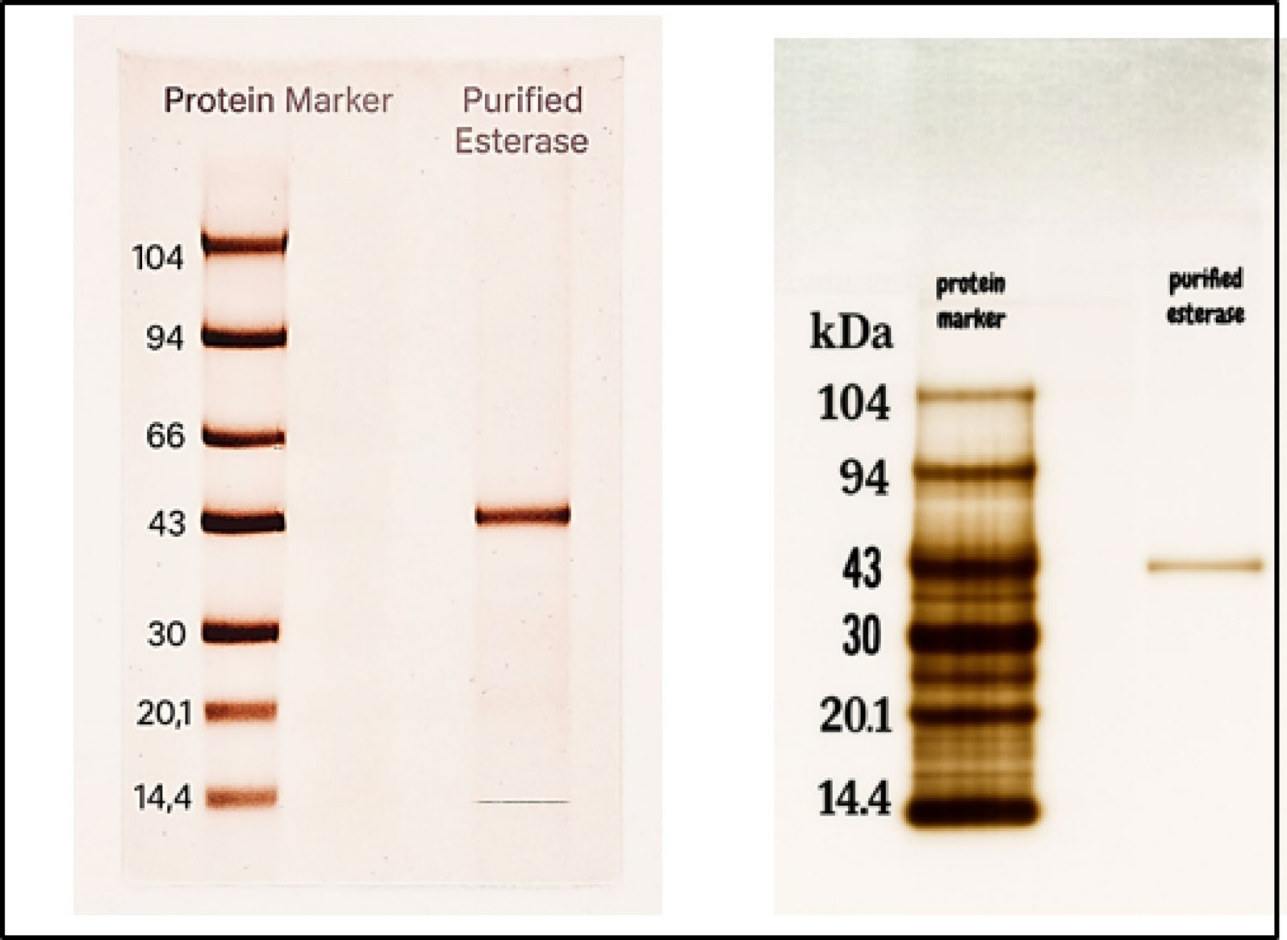
SDS-PAGE of purified esterase from *T*. *afroharzianum* stained with silver nitrate. The first lane is the protein marker ranging from 14 to 104 kDa, Fisher bioreagents, and the second lane is the purified esterase isolated from *T*. *afroharzianum*

When proteins are exposed to SDS and a reducing agent that breaks the disulfide bonds necessary for proper folding, this results in the proteins denaturing into linear chains, with a negative charge that intensifies as the chain elongates. The purity and integrity of the esterase enzyme were assessed by an SDS-PAGE analysis. The purified *T. afroharzianum* esterase exhibited a molecular weight of 43 kDa, as determined by Sephacryl S300 HR with standard proteins, corroborated by SDS-PAGE electrophoresis, as shown in (Fig. 6). The first well of gel is a protein marker lane ranging from 14 to 104 kDa, obtained from Fisher bioreagents, the second lane is purified esterase after gel filtration chromatography (GFC) using Sephacryl S300 HR, which showed a single band, confirming its high purity. Overall, the SDS-PAGE results indicate that the esterase enzyme was effectively purified through gel filtration chromatography and 60% ammonium sulfate fractionation and that the molecular weight obtained via the Sephacryl S300 HR GFC method was precise. The previous studies showed that two distinct esterase enzymes were extracted from the seeds of *Jatropha curcas L.*, with molecular weights of 23.5 kDa and 30.2 kDa as revealed after the SDS-PAGE process [48]. The enzyme esterase isolated from *Trichoderma ressi* showed a molecular weight of 45 kDa [47].

The fungus *Trichoderma* produces an esterase enzyme with a molecular weight of 48 kDa [49]. The glucuronoyl esterase, with a molecular mass of 58 kDa, was uniformly extracted [50]. *Aspergillus nomius HS-1* produced carboxyl esterase with a molecular mass of 60 kDa [51]. Whereas the marine fungus *Fusarium sp*. synthesized esterase with a molecular weight of 76 kDa [52].

### Biochemical characterization of the pure T. afroharzianum

#### Effect of temperature and different values of pH

A key variable influencing esterase activity is temperature. Fluctuating temperatures can alter the conformation of an enzyme, hence affecting its catalytic efficiency. When the enzyme’s activity exceeds the optimum temperature [53].

**Fig. 7:**
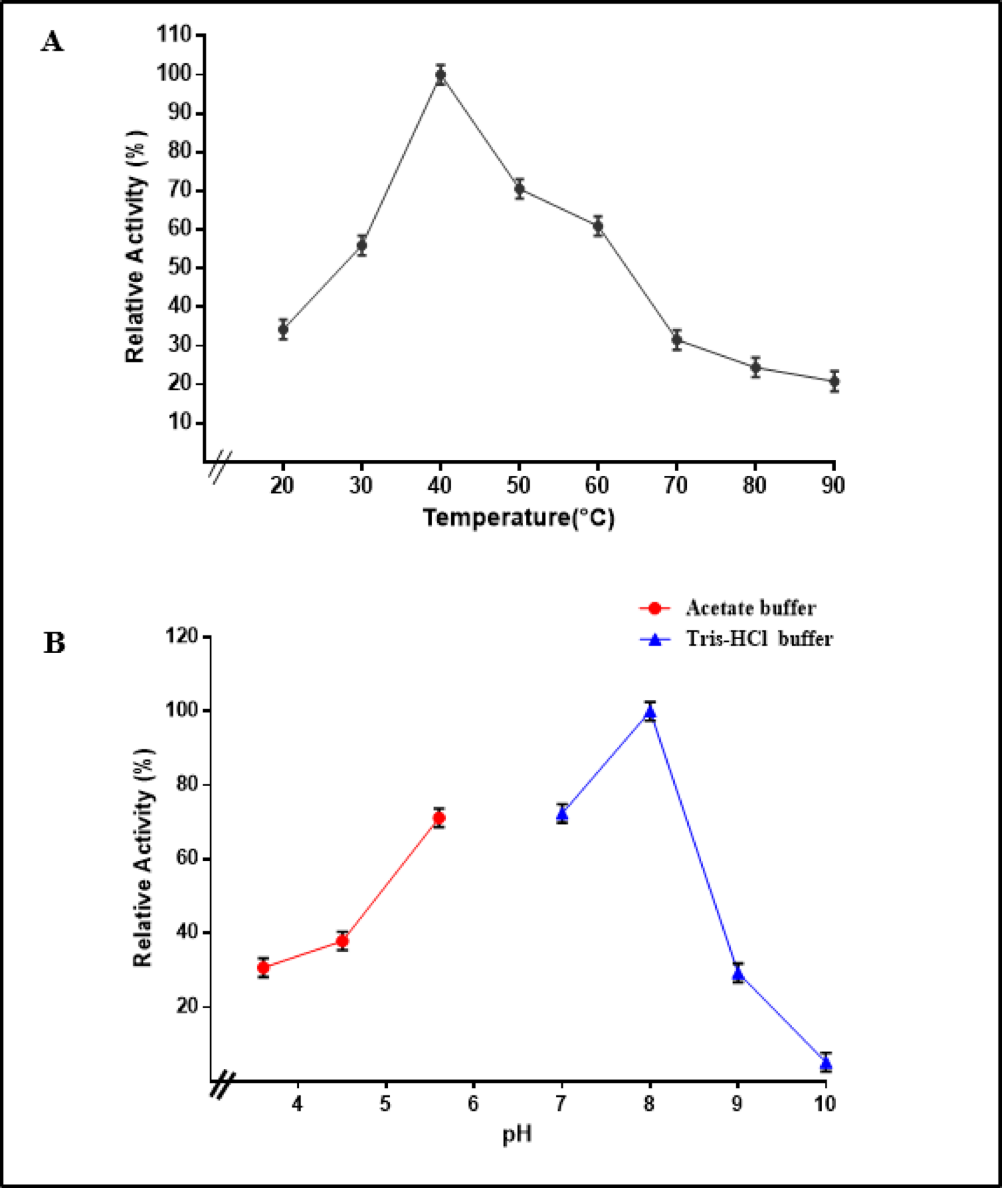
**A**. Effect of temperature on the activity of pure esterase from T. afroharzianum, **B**. Effect of different pH values on *T. afroharzianum* esterase

The enzyme purified from *T. afroharzianum* pure esterase exhibited a significant increase in activity, achieving its maximum at 40 °C, which is the optimal temperature for isolated esterase activity. However, as the temperature increases further, the enzyme activity begins to decrease, indicating that high temperatures lead to enzyme degradation or reduced efficiency, as shown in (Fig. 7A). The previous studies have clarified that the esterase enzyme extracted from *Bacillus spp* indicated that optimal conditions were established at a temperature of 37 °C [34]. The esterase enzyme sourced from *Deinococcus radiodurans* showed maximum catalytic activity at 37 °C [54]. The enzymatic function of esterase Est804 from the metagenomic library significantly declines when temperatures rise above 45 °C [55]. The esterase enzyme derived from *Serratia sp* showed maximum enzyme activity at 50 °C [38].

The pH significantly influences the structure and activity of esterase, making it a critical variable. The enzyme becomes inactive and ceases its function when the pH exceeds a specific range. For enzymes to perform their biological functions optimally, it is essential to supply them with the appropriate pH conditions [56]. Moreover, the buffer solutions, Tris-HCl, acetate, were employed in measuring esterase activity across a range of pH values from 3.6 to 10.

The greatest esterase activity was found in Tris-HCl buffer at pH 8 with 100% relative activity as indicated in (Fig. 7B). This indicates that the enzyme’s optimal pH is slightly alkaline, which is characteristic of most microbial esterases that exhibit peak activity at or above neutral pH levels. This aligns with most microbial esterases that exhibit the best activity within the neutral to alkaline pH spectrum, especially in Tris-based buffers, which maintain enzyme structural stability at elevated pH levels [57]. Also, this is in accordance with the findings of a study that indicated that the greatest esterase activity was found at a solution pH of 8.0 [55]. Esterase activity was significantly elevated between pH 6.0 to 8.0 in the identification and characterization of a novel esterase from *Thauera sp* [58].

A previous study showed that the peak esterase activity was attained with Tris-HCl buffer at pH 8, achieving 29.16 U/mg. Conversely, activity dropped markedly with phosphate buffer at pH 7.0, yielding a specific activity of 17.65 U/mg, which represents 61% of the peak activity. These suggest that esterase showed peak activity with alkaline pH range buffers and very minimal activity with acidic pH range buffers. Buffers are used to regulate and maintain the target pH throughout the enzyme assay, and the interaction of inorganic ions can sometimes facilitate the binding of a substrate molecule to the enzyme’s active site [45] .

The highest specific activity of isolated esterase from *Bacillus licheniformis* in a 0.1 M Tris-HCl buffer at pH 8.0 [45]. Buffers help in adjusting and stabilizing the required pH during the enzyme assay, and the interplay of inorganic ions can sometimes facilitate a substrate molecule’s ability to find or attach to the enzyme’s active site. Elevated pH significantly decreased the enzyme activity as a result of alterations in the enzyme’s spatial structure and conformation. Enzymes reach peak activity at their ideal pH, but alterations in pH can denature the enzyme, leading to a decline in activity [21].

An additional prior study with esterase isolated from *Bacillus pumilus* revealed that peak enzyme activity occurred in 0.05 M Tris-buffer at a pH of 8.0 [59]. Esterase activity of *Psychrobacter cryohalolentis* K5 was maximized in a Tris-HCl buffer at a pH of 8.5 [60]. Furthermore, the optimum condition of esterase enzyme isolated from *Deinococcus* was at pH 8 [54].

The esterase enzyme isolated from *T. afroharzianum* AUMC 16,433 is highly promising because it has optimum standard conditions, which makes this enzyme even more beneficial from an industrial perspective.

### Determination of the kinetic parameters (Km and vmax values)

**Fig. 8:**
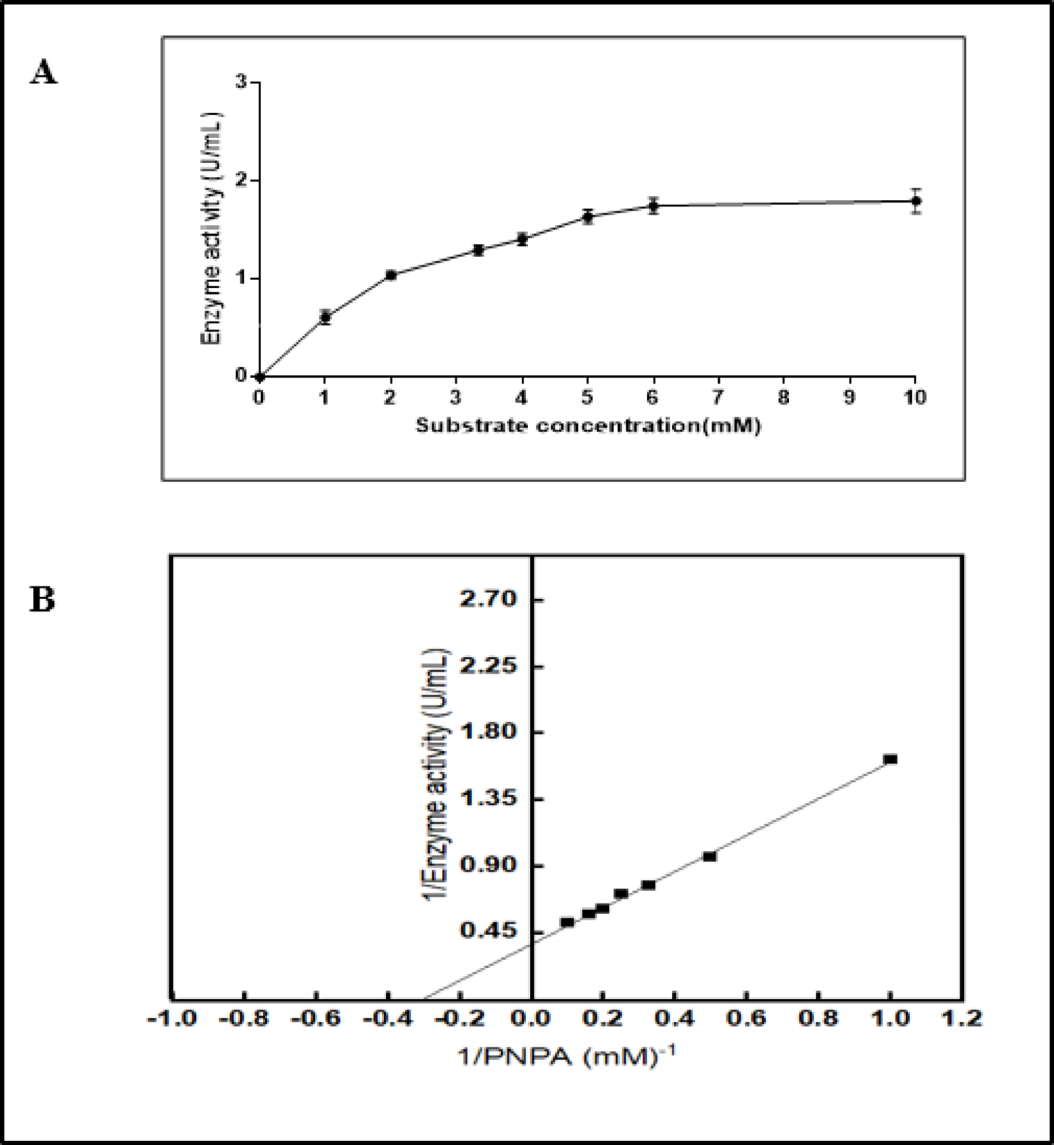
**A**. Different substrate concentrations on pure T. afroharzianum, **B**. Line weaver-burk plot for *T*. *afroharzianum* purified esterase under a series of p-NPA concentrations

The findings elucidate that the optimum enzymatic activity was reached at a substrate concentration of 5 mM, as shown in (Fig. 8A). Moreover, as shown in (Fig. 8B). The kinetic characteristics of esterase isolated from *T. afroharzianum* AUMC 16,433 demonstrated a high affinity (Km) for its substrate p-NPA, which was equal to 3.33 mM, and its maximum velocity Vmax was 2.717 U/mL. A previous study observed that the esterase isolated from *Shewanella sp* exhibited a maximum velocity (Vmax) of 550 U·mg⁻¹ and a Michaelis-Menten constant (Km) of 12.6 mM by p-NPA hydrolysis reaction [61]. The optimal concentration of 2.5 mM p-NPA was identified for the activity of the esterase isolated from *Pantoea disperse* [62]. Esterase from *Bacillus subtilis* DSM402 (BS2), exhibited a Km value of 119 mM for p-NPA as a substrate [63]. Previous studies on esterase using a different substrate have shown that esterase extracted from *Bacillus mojavensis* isolate TH309 exhibited apparent Km and Vmax values of 1.28 mM and 23.88 µmol/min, respectively [31].

### Half-life span

**Fig. 9:**
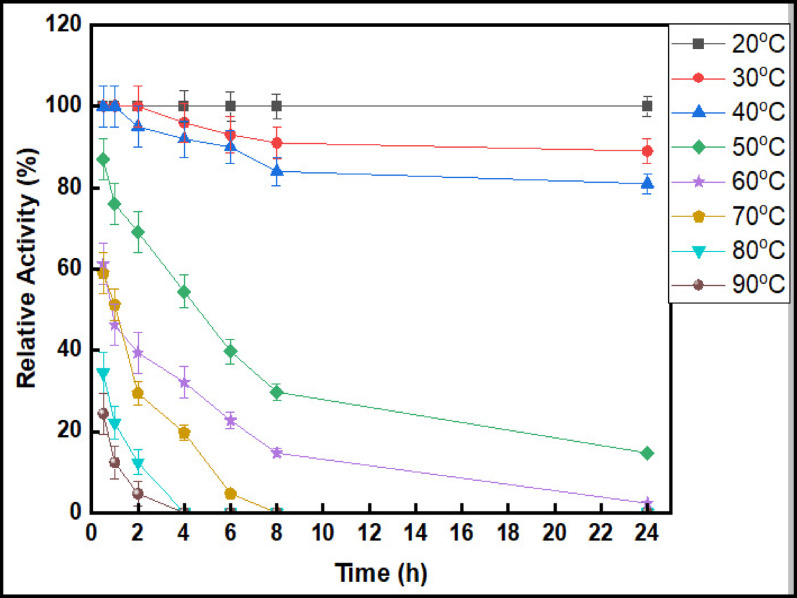
Half-life span of purified esterase isolated from *T*. *afroharzianum*

To demonstrate the enzyme’s ability to withstand harsh conditions at high temperatures. The purified esterase from *T. afroharzianum* half-life span was 54.4% at 50 °C after 4 h, and the enzyme still retained 14.7% of its activity after 24 h at 50 °C. Also, it lasted for 6 h at 70 °C, as shown in (Fig. 9). Previous studies showed that the purified esterase exhibited a half-life of 70 min at 60 °C and 31 min at 80 °C [63]. After a one-hour incubation, the esterase from *Lactobacillus crispatus* exhibited 60% and 10% residual activity at 60 and 65 °C, respectively [64]. Like the esterase extracted from *Trichoderma reesei*, most enzymes demonstrate significant reductions in activity after incubation at 60 °C for 0.5 h [65].

After 4 h of incubation at 60 °C, the esterase from *B. pumilus* SK52.001 exhibited significant thermal stability, retaining 60% residual activity [66]. Notably, the esterase from *Penicillium piceum* has superior thermal stability, with a duration of 220 min at 70 °C and 150 min at 60 °C [67]. The *Aspergillus niger* esterase enzyme exhibited stability at 70 °C for one hour. Following incubation at 80 °C for one and four hours, the enzyme activity diminished to 56% and 10%, respectively [42] .

### Dye decolorization application

Synthetic dyes are in significant demand, particularly within the textile and fabric production sectors, many of which pose risks to environmental and human health. Synthetic dyes have been reported to possess mutagenic and carcinogenic potential. Enzymatic, chemical, and microbiological treatments have been employed to mitigate their adverse effects before environmental release. Esterases enzymes are innovative biocatalysts that are significant in biotechnological processes [68].

**Fig. 10:**
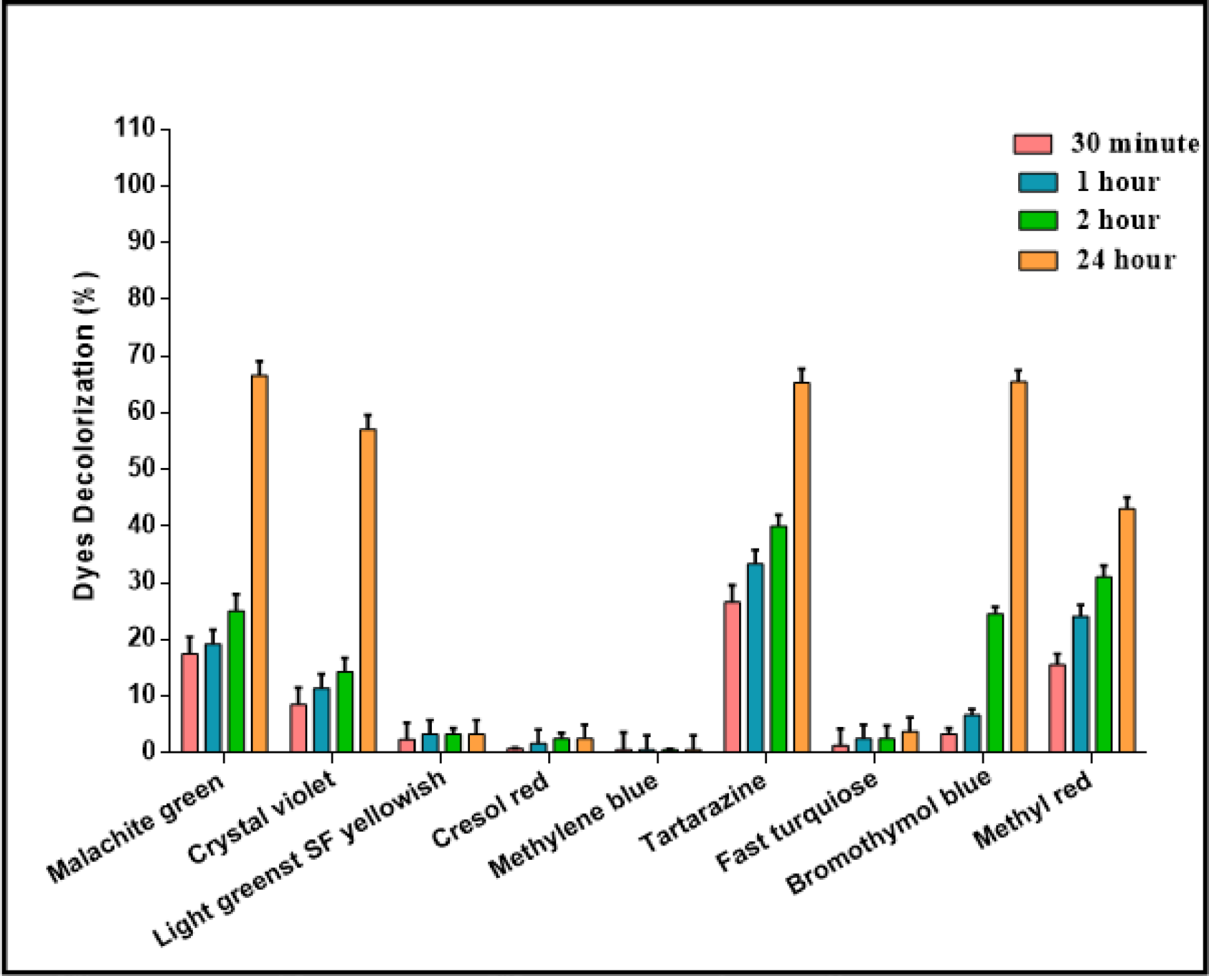
Synthetic dye decolorization impacts using biodegredable purified *T. afroharzianum*

Below the optimum conditions of pH 8 and 50 °C, the purified esterase obtained from *T. afroharzianum* was incubated with various dyes was monitored at different time intervals (30 min, 1 h, 2 h, and 24 h) as shown in (Fig. 10). Our results indicated that malachite green, bromothymol blue and tartrazine were rapidly decolorized, reaching the highest extent of 66%, 65.5% and 65.3% respectively, after 24 h incubation, and the solution became significantly less dense as shown in supplementary file (Fig. S2). Achieved decolorization rates of crystal violet and methyl red exhibited moderate decolourization, becoming lighter in hue, with degradation rates of 57.1% and 43.1%, respectively. It is the first time to study the use of *T. afroharzianum* esterase to decolorize these dyes. This highlights the importance of using esterase enzyme in future studies to decolorize several types of dyes, particularly those used in this study. While, Direct fast turquoise blue, light green SF yellowish and Cresol red showed low and poor decolorization rates within 24 h with 2.5%, 3.3% and 3.75%, respectively. Meanwhile, methylene blue dye exhibited minimal alteration, with the lowest depolarization rate of 0.6% after 24 h, maintaining most of its original hue and appearing darker than others, indicating this dye was less susceptible to decolorization by the esterase enzyme. A common catalytic triad Ser-His-Asp is crucial in ester hydrolases, where the serine residue is the nucleophile to attack the carbonyl carbon of the scissile bond [69]. Thus, this enzyme can destroy the dye without an ester linkage [70].

Malachite green (MG) is a dark green crystalline solid and a water-soluble cationic basic dye, which is chemically identified as N-methylated diamino triphenylmethane. It is one of the most widely used dyes in various industries such as textiles, papermaking, pharmaceuticals, and cosmetics. Several reports have pointed out its cytotoxicity against cells of different organisms, including humans. Besides this, MG has also been reported for its carcinogenic, teratogenic, and mutagenic effects on human cells. Esterase enzyme extracted from *Bacillus. spp*, exhibited decolorization capabilities for triphenylmethane dyes, such as malachite green, with 17.18% [34]. Moreover, the esterase from the *Salinicoccus roseus* RF1H strain had promising decolorization results with the synthetic dyes malachite green, Congo red, brilliant yellow, and orange G [71]. In our study, this is the first time to suggest the decolorization mechanism of *T. afroharzianum* esterase on MG dye as this dye exhibited superior decolorization efficiency because its chromophore structure requires only two demethylation reactions for decolorization as showed in supplementary file (Fig. S3). This simplified decolorization pathway contrasts with other dyes that possess multiple substituents and require more extensive processing steps, resulting in longer treatment times and reduced efficiency [72, 73]. Moreover, the structure of malachite green is particularly susceptible to nucleophilic attack, leading to efficient cleavage of chromophore bonds and formation of less toxic aromatic intermediates [74, 75].

Crystal violet is also another triphenylmethane dye that finds extensive use in bacterial identification, dyeing plastics, waxes, paints, and inks. It is utilized as an antibacterial agent in poultry feed as well as in differential culture medium but is toxic upon dermal absorption, with the capacity to irritate the eyes, skin, and respiratory tract [76]. Moreover, malachite green was decolorized faster than crystal violet [77], and this was confirmed in our result, where the isolated pure esterase enzyme had a greater ability to decolorize malachite green than crystal violet, despite both being triphenylmethane. The suggested mechanism this is that to decolorize crystal violet, it is essential to demethylate the three dimethyl groups, known as chromophore groups, as observed in the supplementary file (Fig. S4). Conversely, the decolorization of malachite green necessitates merely two demethylations [78]. Dyes with a higher number of substituents necessitate more processing time [73].

Tartrazine (TAR) dye is a carcinogenic and mutagenic lemon-yellow azo dye that is primarily used in the food industry and is released into waste streams. It has several adverse effects, including anxiety, headache, depression, visual impairment, asthma, itching, weakness, and suffocation [79]. The suggested decolorization mechanism is that esterase enzyme adding water molecules and undergo neutrophilic attack to the aromatic structure of dye as shown in supplementary file (Fig. S5).

Bromothymol blue (BTB) is a sulfonephthalein derivative of textile dyes. It is yellow in acid, green in neutrality, and blue in basicity because of its halochromic nature. The chemical is extensively employed as a biological stain and acid-base indicator. BTB, even upon usage, can cause eye, skin, and respiratory tract irritation [80]. The suggested decolorization mechanism is that esterase enzyme adding water molecules and undergo many resonances to the aromatic rings bonds and remove the chromophore groups using the neutrophilic attack as shown in supplementary file (Fig. S6).

Methyl red (MR) is a mono-azo dye with harmful, mutagenic, and carcinogenic properties and is, therefore, a significant water pollutant [81]. The complex breakdown of methyl red in liquid waste poses different hazards to living organisms. Due to its non-biodegradability and toxicity, the elimination of methyl red from wastewater is critical [82]. Esterase is an enzyme that breaks ester linkages but also acts on structurally similar functional groups, such as amides [83]. Methyl red contains an amide group [84], then esterases are responsible for its decolourization by breaking the amide linkage bond.

Consequently, our findings validated that the catalytic dye decolorization efficacy of the examined esterase from *T. afroharzianum* strain AUMC 16,433 enables its extensive use in various environmental bioremediation efforts.

Cresol red (CR), also known as o-cresol red, belongs to the phthalein and sulphonphthalein group of dyes. which are potentially carcinogenic or mutagenic [85]. CR has a high molecular weight and incorporates oxygen and sulphur atoms as well as aromatic rings in its structure; these qualities indicate it is a viable corrosion inhibitor [86]. Previous study observed that *Trichoderma harzianum* M06 enzymes were capable of decolorizing cresol red [87] .

Phthalocyanine dye, also known as fast turquoise, is a copper-containing organic compound. It consists of four iso-indole units connected by a nitrogen-containing ring. Its poor decolorization effectiveness is due to the inability to dissolve the metal-ion coordination bond [88].

Methylene blue (MB) is a thiazine dye that has three linear benzene rings without side chains. Thus, MB is a basic cationic dye with positively charged polymethine with an amino autochrome unit. It is poisonous and carcinogenic. Large amounts of it are released into groundwater and surface water after industrial use. MB dye’s monoamine oxidase-inhibiting properties can cause lethal serotonin poisoning in humans and aquatic wildlife [89].

Light green SF yellowish is also known as acid green, green SF, food green 2, and acid brilliant green. Usually as a disodium salt [90]. It is used to diagnose human medical cells and examine human samples histologically and cytologically.

No previous studies have been conducted on the use of esterases to decolorize methylene blue, fast turquoise, light green SF-yellowish, and cresol red dyes. The results of this current study also show that the ability of esterases to decolorize these dyes is poor, with decolorization rates in 24 h with 0.6%, 2.5%, 3.3%, and 3.75%, respectively. The suboptimal decolorization outcomes mostly stem from the incompatibility between the esterase enzymatic mechanism (hydrolysis and nucleophilic attack) and the chemical features of these dyes, which necessitate distinct enzymatic actions (oxidation/reduction) that are unrelated to bond hydrolysis.

## Conclusion

Bioremediation techniques for the decolorization of hazardous dyes represent a significant area of research interest. This study optimized the endophytic *T. afroharzianum* strain AUMC 16,433 to establish ideal fermentation parameters for esterase production.The *T. afroharzianum* pure esterase was observed as a single protein band using SDS-PAGE with a molecular weight of 43 kDa, indicating that this enzyme is monomeric. Additionally, half-life span stability was 54.4% at 50 °C after 4 h, and the enzyme still retained 14.7% of its activity after 24 h at 50 °C; it remained mainly stable even after 24 h at that temperature. Also, esterase was highly active at pH 8. The peaked esterase decolorization efficiency after 24 h was for malachite green, tartrazine and bromothymol blue dyes. Thus, this study according to our knowledge is considered the first microbial thermostable production of esterases from fungal endophyte *T. afroharzianum* strain AUMC 16,433, that isolated from the *cladodes* of *Opuntia ficus-indica*. Also, it recommended as environmentally friendly biocatalysts, in line with the global movement towards efficient and sustainable industrial practices.

## Future prospective

Future research should emphasize the immobilization of esterase enzymes derived from the endophytic *T. afroharzianum* strain AUMC 16,433. Future research should assess the dye decolorization efficacy of these immobilized enzymes utilizing real industrial effluent samples. Furthermore, studies should evaluate the reusability capability of immobilized enzymes across various environmental conditions to determine their practical practicality for large-scale industrial use.

## Supplementary Information

Below is the link to the electronic supplementary material.

Supplementary Material 1

## Data Availability

The data was available from the corresponding author upon request.

## Abbreviations

GFC: Gel filtration chromatography
p-NPA: P-nitrophenyl acetate
SDSPAGE: Sodium dodecyl sulfate-polyacrylamide gel electrophoresis
CCD: Central composite design
BLAST: Basic local alignment search tool
Km: Michaelis-Menten constant
Vmax: Maximum reaction velocity
ANOVA: Analysis of variance
PDA: Potato dextrose agar
TMED: Tetramethyl ethylenediamine
RSM: Response Surface Methodology
MG: Malachite green
MB: Methylene blue
BTB: Bromothymol blue
TG: Tartrazine dye
