# Supplementary material for: Production, optimization and characterization of esterase isolated from a new endophytic Trichoderma afroharzianum strain AUMC 16,433 and its applications in dye decolorization
Source: Microb Cell Fact. 2025 Sep 9;24:201. doi: 10.1186/s12934-025-02832-8 (PMC12418644; doi:10.1186/s12934-025-02832-8)
Supplement: Supplementary file 1 — Supplementary Material 1 [file 12934_2025_2832_MOESM1_ESM.pdf]

Supplementary file

Production, optimization and characterization of esterase isolated from a new endophytic *Trichoderma afroharzianum* strain AUMC 16433 and its applications in dye decolorization

Yehia A.-G. Mahmoud[1], Nisrin S. Alamin[2], Tarek M. Mohamed[2], Nesma A. El-Zawawy[1] and Maha M. Salem[2*].

[1] Botany and Microbiology Department, Faculty of Science, Tanta University, 31257, Tanta, Egypt.
[2] Biochemistry Division, Chemistry Department, Faculty of Science, Tanta University, 31257, Tanta, Egypt.

Emails:
yehia.mahmoud@science.tanta.edu.eg
Nisreen_181425_pg@science.tanta.edu.eg
Tarek.ali@science.tanta.edu.eg
nesma.elzawawi@science.tanta.edu.eg
maha_salem@science.tanta.edu.eg

*Correspondence and proof:

Dr. Maha M. Salem (Ph.D.)

Biochemistry Division, Chemistry Department, Faculty of Science, Tanta University, 31257, Tanta, Egypt.

Email: maha_salem@science.tanta.edu.eg

Table S1. Experimental variables for Placket-Burman at different levels.

Factors	Variables	Units	Experimental values	
			Low level (-1)	High level (+1)
A	Cotton seed oil	mL	3	5
B	Peptone	Gram	1	2
C	Maltose	Gram	0.5	1.5
D	pH	-	7	9
E	Temperature	◦C	28	40
F	Inoculum size	-	0.5	1.5

Table S2. Experimental variables for central composite design at different levels**.**

Factors	Variables	Units	Experimental values		
			Low level (-1)	Intermediate level (0)	High level (+1)
A	Cotton seed oil	mL	3	4	5
B	Peptone	Gram	1	1.5	2
C	Maltose	Gram	0.5	1	1.5

Table S3. Synthetic dyes were used in this study to estimate the ability of esterase for dye decolorization.

Dyes	Molecular Weight (g/mol)	chemical structure
Malachite green (εmax= 617 nm)	364.91	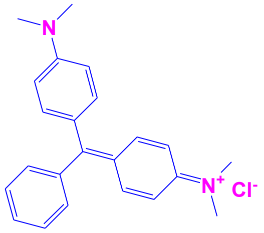
Methyl red (εmax= 410 nm)	269.30	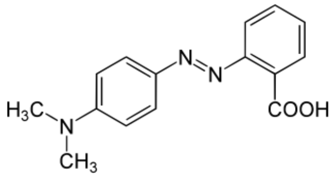
Fast turquoise blue (εmax= 550 nm)	780.17	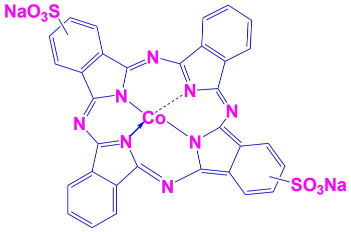
Methylene blue (εmax= 665 nm)	319.85	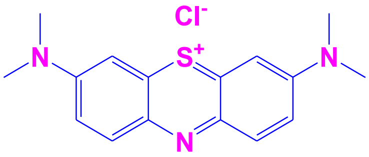
Crystal violet (εmax= 590 nm)	407.99	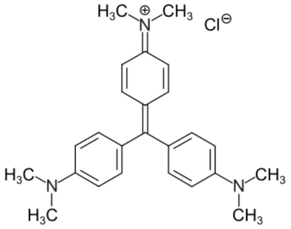
Bromothymol blue (εmax= 460 nm)	624.4	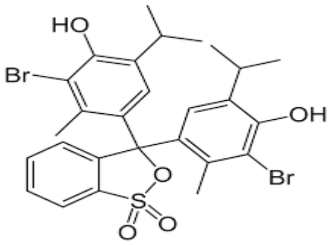

Light green SF yellowish<br><br>(ε_{max}= >600 at 631-634 nm in water)	749.893	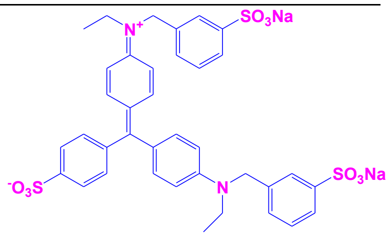
Tartarazine<br><br>(ε_{max}= 435-500 at 425-429 nm in water)	534.3	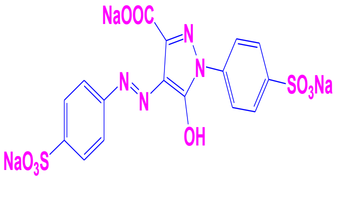
Cresol red<br><br>(ε_{max}= 442.01 -590.16 nm)	404.41	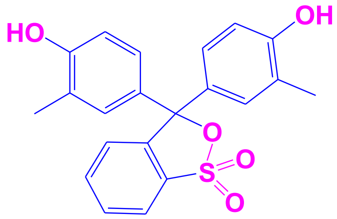

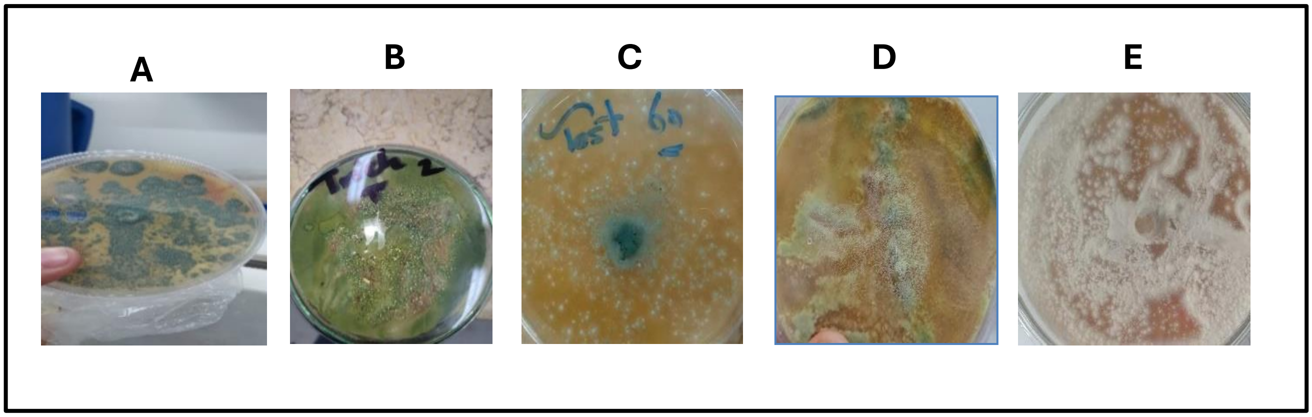

Figure S1. Fungal endophytes were isolated from *O. ficus-indica* and screened for esterase activity A. EF-1, B. EF-2, C. EF-3, D. EF-4 and E. EF-5 strain were used in this study.

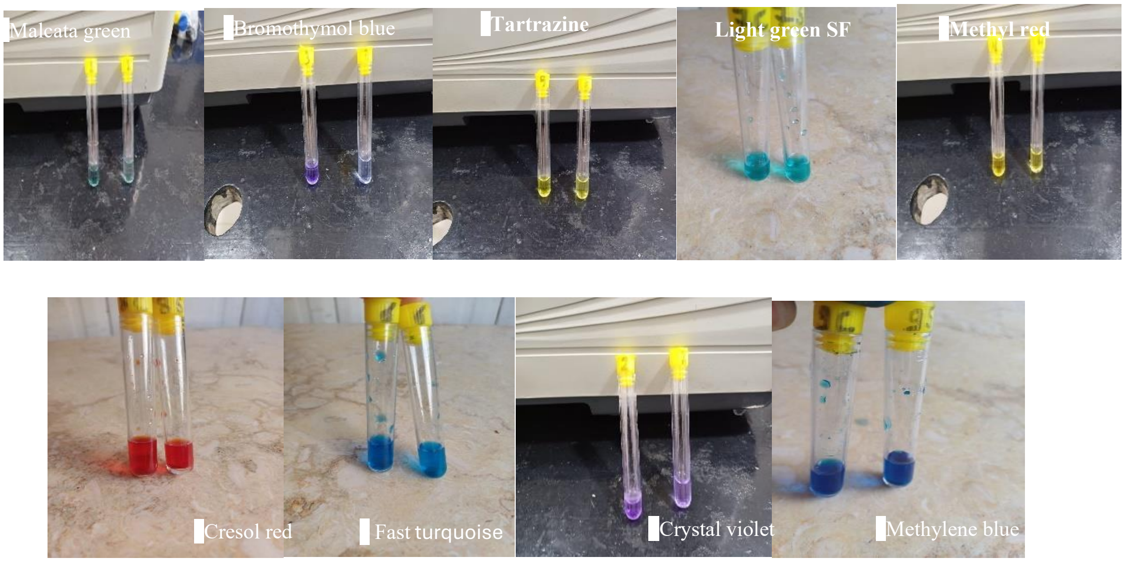

Figure S2. The image of the dye sample before and after 24 hours of treatment with purified esterase enzyme.

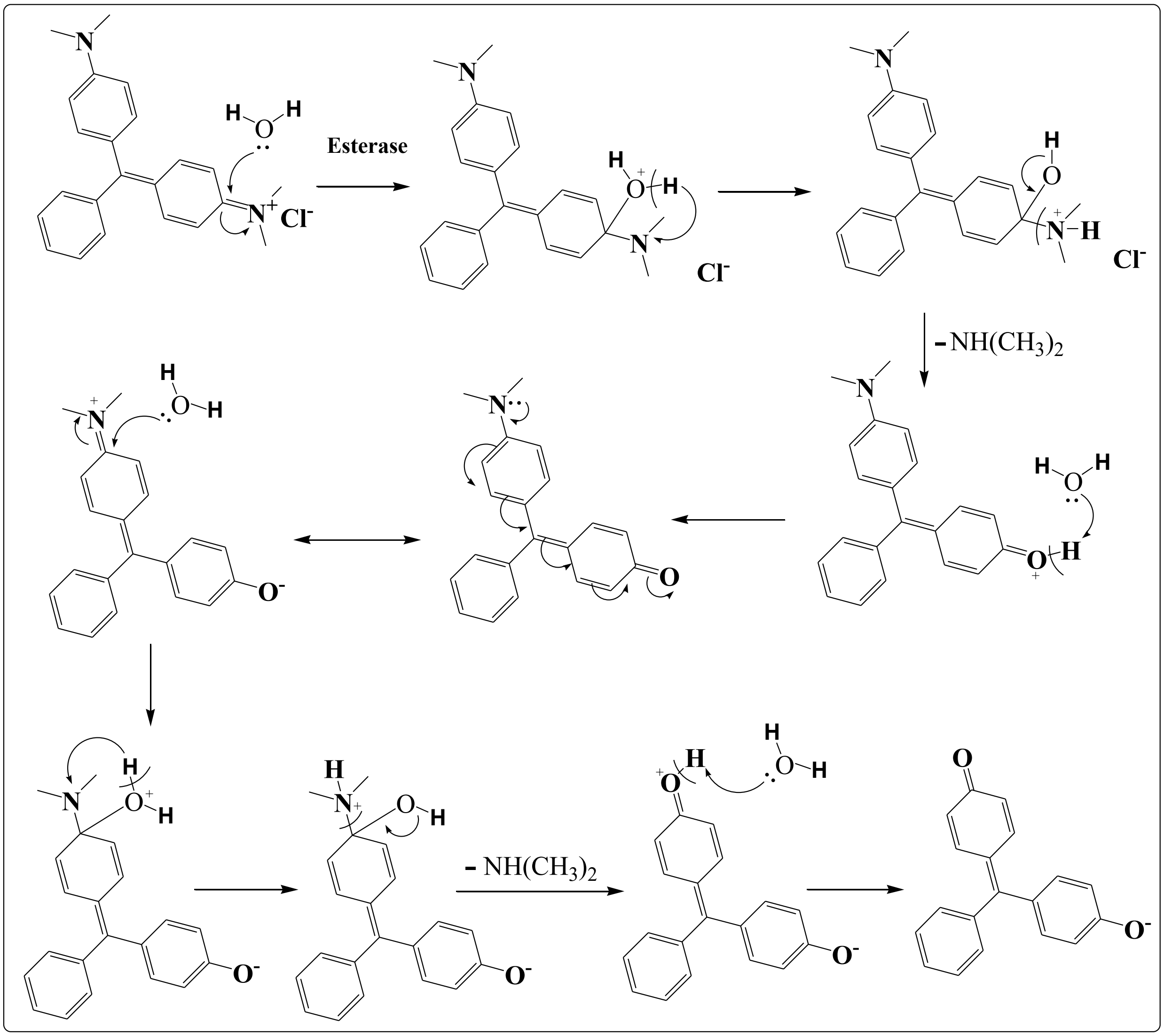

Figure S3: The suggested mechanism of malachite green dye decolorization by *T. afroharzianum* esterase.

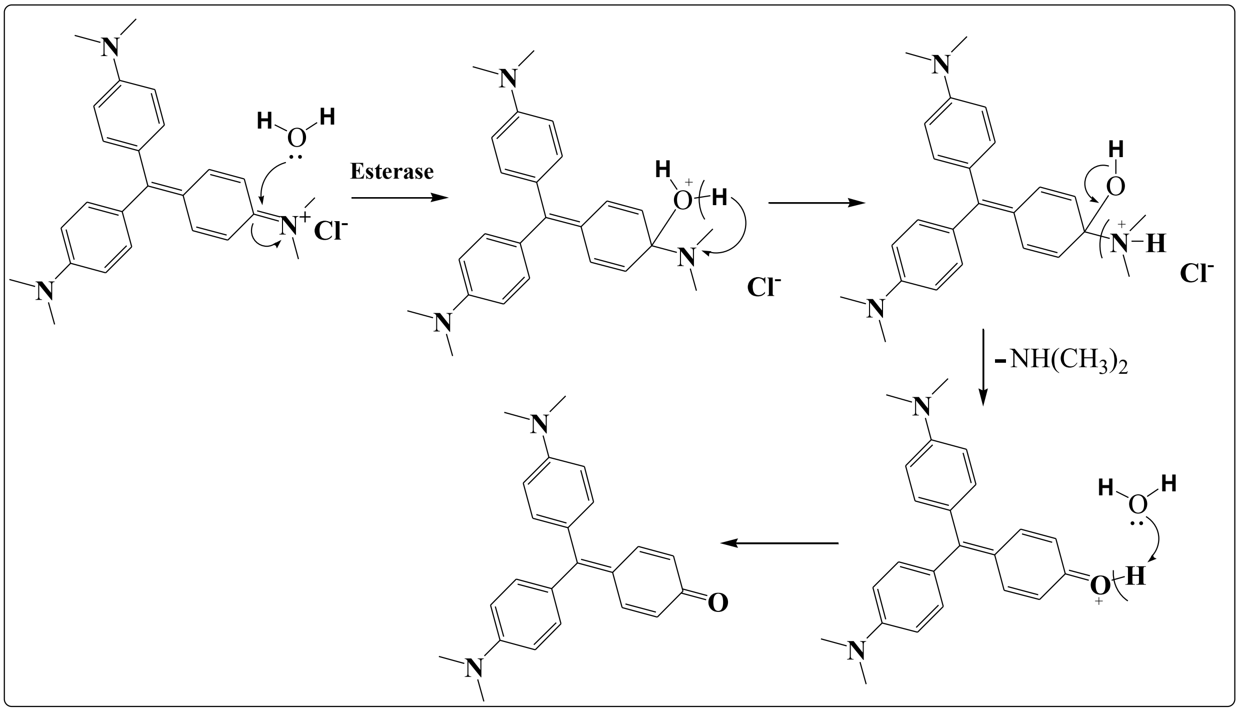

Figure S4: The suggested mechanism of crystal violet dye decolorization by *T. afroharzianum* esterase.

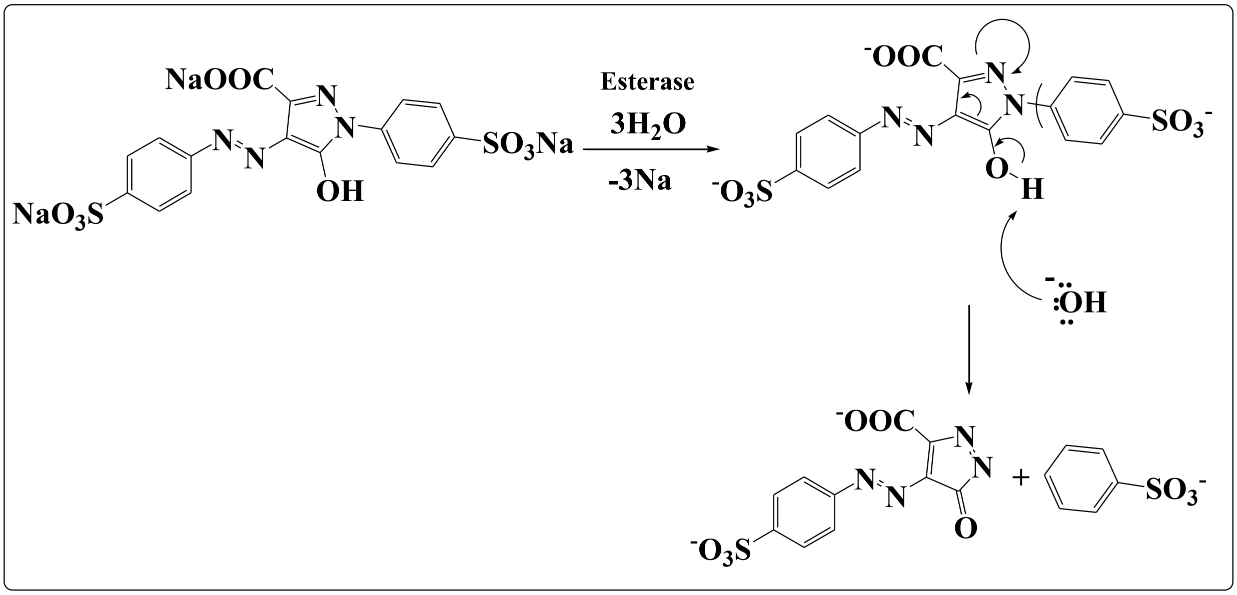

Figure S5: The suggested mechanism of tartrazine dye decolorization by *T. afroharzianum* esterase.

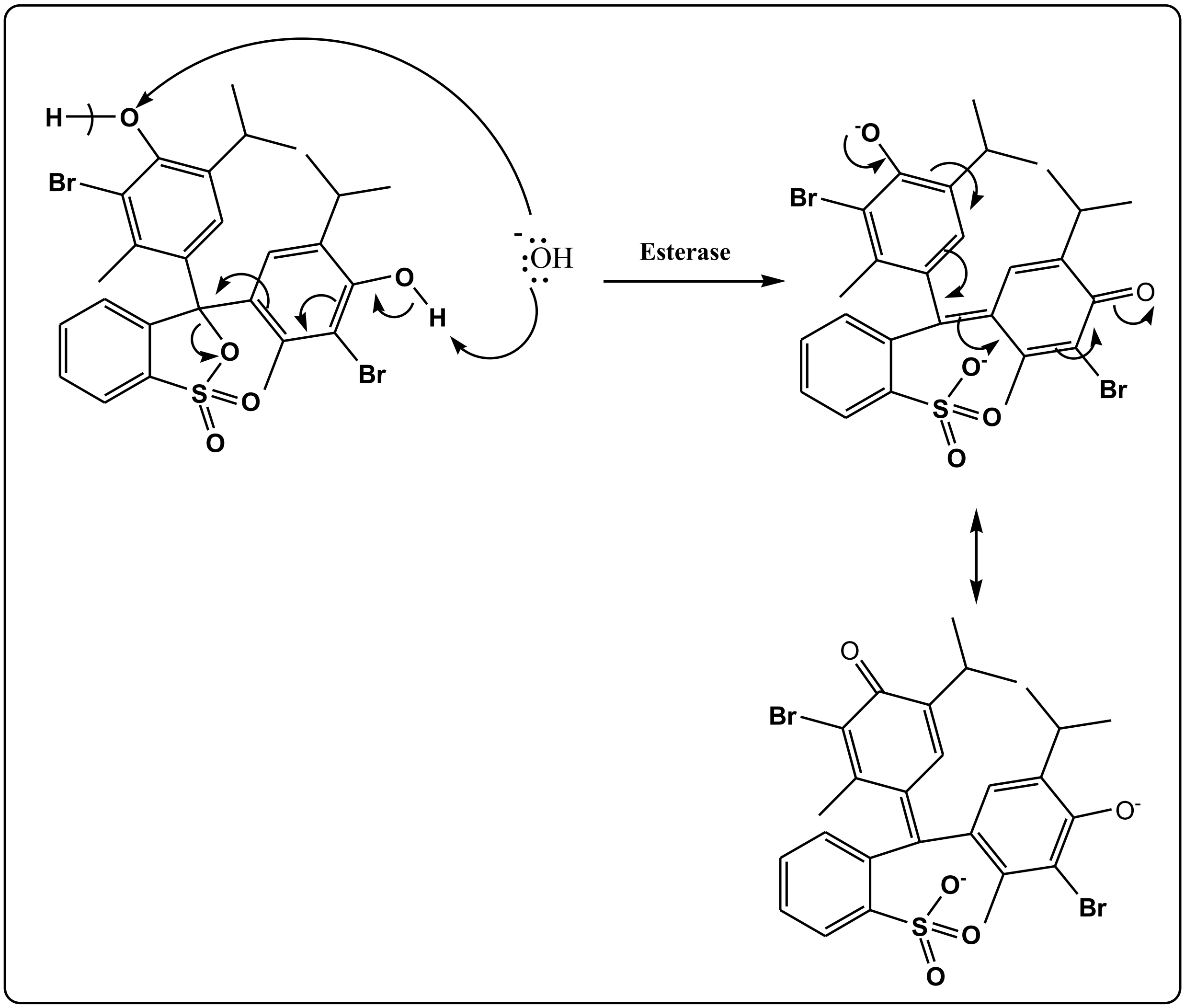

Figure S6: The suggested mechanism of bromothymol blue dye decolorization by *T. afroharzianum* esterase.
